# High field magnetometry with hyperpolarized nuclear spins

**DOI:** 10.1038/s41467-022-32907-8

**Published:** 2022-09-19

**Authors:** Ozgur Sahin, Erica de Leon Sanchez, Sophie Conti, Amala Akkiraju, Paul Reshetikhin, Emanuel Druga, Aakriti Aggarwal, Benjamin Gilbert, Sunil Bhave, Ashok Ajoy

**Affiliations:** 1 Department of Chemistry, University of California, Berkeley, Berkeley, CA USA; 2 Energy Geoscience Division, Lawrence Berkeley National Laboratory, Berkeley, CA USA; 3 OxideMEMS Lab, Purdue University, West Lafayette, IN USA; 4 Chemical Sciences Division, Lawrence Berkeley National Laboratory, Berkeley, CA USA

**Keywords:** Quantum metrology, Solid-state NMR

## Abstract

Quantum sensors have attracted broad interest in the quest towards sub-micronscale NMR spectroscopy. Such sensors predominantly operate at low magnetic fields. Instead, however, for high resolution spectroscopy, the high-field regime is naturally advantageous because it allows high absolute chemical shift discrimination. Here we demonstrate a high-field spin magnetometer constructed from an ensemble of hyperpolarized ^13^C nuclear spins in diamond. They are initialized by Nitrogen Vacancy (NV) centers and protected along a transverse Bloch sphere axis for minute-long periods. When exposed to a time-varying (AC) magnetic field, they undergo secondary precessions that carry an imprint of its frequency and amplitude. For quantum sensing at 7T, we demonstrate detection bandwidth up to 7 kHz, a spectral resolution < 100mHz, and single-shot sensitivity of 410pT$/\sqrt{{{{{{{{\rm{Hz}}}}}}}}}$. This work anticipates opportunities for microscale NMR chemical sensors constructed from hyperpolarized nanodiamonds and suggests applications of dynamic nuclear polarization (DNP) in quantum sensing.

## Abstract (web summary)

Quantum sensors based on NV centers in diamond find applications in high spatial resolution NMR spectroscopy, but their operation is typically limited to low fields. Sahin et al. demonstrate a high-field sensor based on nuclear spins in diamond, where NV centers play a supporting role in optical initialization.

## Introduction

**Fig. 1:**
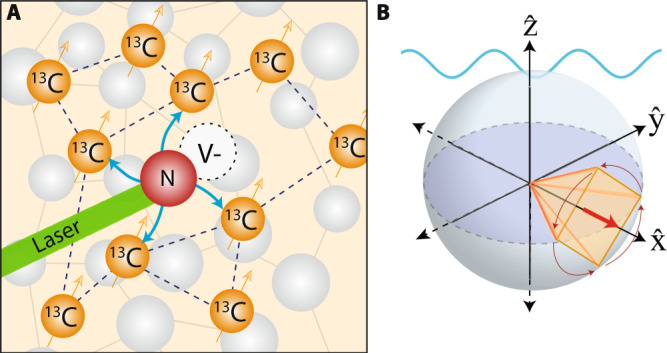
^13^C Sensor Strategy. **A** System. Diamond lattice with dipolar coupled (dashed lines) ^13^C nuclei, hyperpolarized (blue arrows) by optically pumped NV center defects. **B** Principle. Hyperpolarized ^13^C nuclei are driven in to the $\hat{{{{{{{{\bf{x}}}}}}}}}$ axis (red arrow) via spin-locking. When an AC field (blue) is applied along $\hat{{{{{{{{\bf{z}}}}}}}}}$, the spins undergo secondary precessions (arrows). Deviation from $\hat{{{{{{{{\bf{x}}}}}}}}}$ constitutes the magnetometer signal. Case shown corresponds to *θ* = *π*/2 (Fig. 2A), where the spins trace the corners of a square when projected onto the $\hat{{{{{{{{\bf{y}}}}}}}}}-\hat{{{{{{{{\bf{z}}}}}}}}}$ plane over four pulses (period 4*τ* in Fig. 2A).

The discrimination of chemical analytes with sub-micron scale spatial resolution is an important frontier in nuclear magnetic resonance (NMR) spectroscopy^1–3^. Quantum sensing methods have attracted attention as a pathway to accomplish these goals^4^. These are typified by sensors constructed from the nitrogen vacancy (NV) defect center in diamond^5,6^—electrons that can be optically initialized and interrogated^7,8^, and made to report on nuclear spins in their environment. However, NV sensors are still primarily restricted to bulk crystals and operation at low magnetic fields (*B*_0_ < 0.3 T)^9–12^. Instead, for applications in NMR, high fields are naturally advantageous because the chemical shift dispersion is larger, and analyte nuclei carry higher polarization^13^. The challenge of accessing this regime arises from the rapidly scaling electronic gyromagnetic ratio *γ*_*e*_, which makes electronic control difficult at high fields^14,15^. Simultaneously, precise field alignment^16^ is required to obtain viable NV spin-readout contrast^17^. The latter has also made nanodiamond (particulate) magnetometers challenging at high fields. If viable, such sensors could yield avenues for "targetable” NMR detectors that are sensitive to analyte chemical shifts^18–20^, and provide a sub-micron scale spatial resolution determined by particle size^2,12,21^. They also anticipate new applications in high-resolution high-field magnetometry in condensed matter systems^22–27^. In this paper, we propose alternate approach towards overcoming these technical challenges. We construct a magnetometer out of hyperpolarized nuclear spins (See Fig. 1): ^13^C nuclei in the diamond serve as the primary magnetic field sensors, while NV centers instead play a supporting role in optically initializing them^28,29^. By combining hyperpolarization, long-lived transverse sensor lifetimes, and their continuous interrogation, we show that nuclear magnetometers can produce sensitivities comparable to traditional electronic (NV center) sensors, but in the high-field regime.

The advantages of ^13^C nuclei as sensors stem from their attractive properties. Their low *γ*_*n*_ ≈ *γ*_*e*_/3000, enables control and interrogation at high fields (*B*_0_ > 1 T). The benign scaling of the ^13^C Larmor frequency allows the scaling to higher magnetic fields to be easier than for NV centers. In contrast to NV electronic spins, transitions of spin-1/2 ^13^C nuclei are determined solely by magnetic fields and not influenced by crystal lattice orientation^29,30^, so they need not be aligned in a specific direction. They have long rotating frame lifetimes ${T}_{2}^{\prime} \sim 90$ s ^31^, orders of magnitude greater than their NV center counterparts. Similarly, their longitudinal lifetimes *T*_1_ > 10 min^32,33^ are long even at modest fields. This can allow a physical separation between field regions corresponding to ^13^C initialization and sensing, and for the ^13^C sensors to be transported between them^33^. ^13^C nuclei can be non-destructively readout via RF techniques^34^, allowing continuous sensor interrogation without reinitialization. This allows real-time tracking of a changing magnetic field for extended periods^35^. RF ^13^C readout is also background-free, i.e., the ^13^C signal from the hyperpolarized diamond is likely orders of magnitude stronger than the surrounding analyte^36^. Moreover, it is immune to optical scattering^37^, permitting deployment in real-world media.

While these properties appear attractive at first glance, nuclear spins have previously been considered ineffective as quantum sensors. The low *γ*_*n*_, while ideal for high field operation, would be expected to yield low sensitivity^4^, and poor state purity (thermal polarization is ≈ 10^−5^ even at 7 T). Strong ( ~ 1 kHz) dipolar coupling between ^13^C nuclei makes Ramsey-like sensing protocols untenable. This results in a rapid free induction decay (FID) $${T}_{2}^{*} < $$2 ms^34^ and limits sensor integration time.

Here, we demonstrate that these shortcomings can be mitigated. We exploit optical hyperpolarization of ^13^C nuclei (Fig. 1A)^28,29,38,39^ and spin-lock readout scheme that suppresses evolution under dipolar interactions^31,40^. The resulting > 10,000-fold extension in ^13^C lifetimes, from ${T}_{2}^{*}\to {T}_{2}^{\prime}$, provides the basis for expanding sensor readout to minute long periods^31^. These long ^13^C rotating-frame lifetimes can at least partially offset sensitivity losses arising from the low *γ*_*n*_. The sensing strategy is described in Fig. 1B. Hyperpolarized ^13^C nuclei are placed along the transverse axis $\hat{{{{{{{{\bf{x}}}}}}}}}$ (red arrow) on the Bloch sphere at high field, where they are preserved for multiple-second long ${T}_{2}^{\prime}$ periods^31^. Any subsequent deviation of the spin state from $\hat{{{{{{{{\bf{x}}}}}}}}}-\hat{{{{{{{{\bf{y}}}}}}}}}$ plane can be continuously monitored and constitutes the magnetometer signal. In the presence of the target magnetic field, ${{{{{{{{\bf{B}}}}}}}}}_{{{{{{{{\rm{AC}}}}}}}}}(t)={B}_{{{{{{{{\rm{AC}}}}}}}}}\cos (2\pi {f}_{{{{{{{{\rm{AC}}}}}}}}}t+{\varphi }_{0})\hat{{{{{{{{\bf{z}}}}}}}}}$ at a frequency *f*_AC_, the nuclei undergo a secondary precession in the $\hat{{{{{{{{\bf{y}}}}}}}}}-\hat{{{{{{{{\bf{z}}}}}}}}}$ plane that carries an imprint of *f*_AC_. Long ${T}_{2}^{\prime}$ yields high spectral resolution.

As opposed to traditional quantum sensing, magnetometry is performed here with nuclear spins already in "coupled-sensor” limit, i.e., in the regime where $\left\langle d\right\rangle {T}_{2}^{*} \sim 1$. The key novelty of our approach is (1) the ability to mitigate this intersensor coupling while simultaneously (2) rendering spins sensitive to external magnetic fields. The protocol we propose achieves these twin aims in a highly robust manner evidenced by the ability to apply > 250,000 pulses to the sensor spins while continuously tracking their evolution. Moreover, unlike conventional quantum sensing, here by measuring amplitude and phase of the sensor response, we are able to discern the sensor position on two quadratures simultaneously on the Bloch sphere.

## Results

### High-field magnetometry with hyperpolarized ^13^C nuclei

**Fig. 2:**
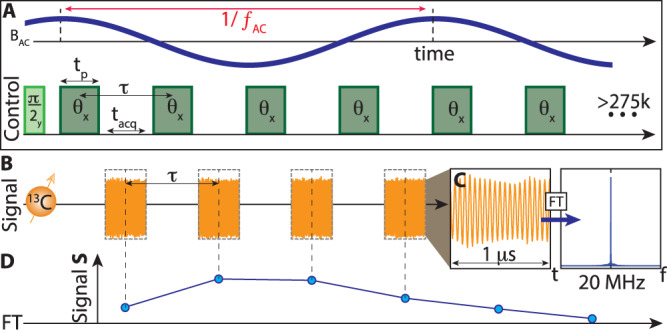
Magnetometry Protocol. **A** ^13^C sensing sequence consists of a train of *θ* flip-angle pulses (green) separated by interpulse period *τ*. Upper trace (blue) representatively (with an arbitrary phase) shows an applied AC field of frequency *f*_AC_; it is denoted here in the "resonant'' configuration for *θ* = 75^∘^. Pulses have width *t*_*p*_, separated by acquisition periods *t*_acq_. **B** Directly observed ^13^C Larmor precession. During *t*_acq_, ^13^C precession is sampled every 1 ns. Orange curves show representative raw data taken in a single-shot for six consecutive readout windows between successive pulses. Here *t*_acq_ = 32 *μ*s, *t*_*p*_ = 30 *μ*s, *τ* = 73 *μ*s, and *f*_AC_ = 2 kHz. **C** Zoom into a 1*μ*s portion of an acquisition window. Fourier transform (blue) displays the heterodyned Larmor precession at *f*_het_ = 20 MHz. **D** Rotating frame signal. Magnitude of signal at *f*_het_ in each window is plotted (blue points), extracting the 20 MHz ± 32 kHz component in **C**. The line joining points shows oscillations whose frequency components reflect harmonics of *f*_AC_ (see Fig. 5). Note here, the AC field schematic does not correlate with the actual phase of the signal.

Experiments here are conducted on a single-crystal diamond, but can be extended to powders. The sample has ~ 1 ppm NV center concentration and natural abundance ^13^C. Hyperpolarization occurs through a method previously described at 38 mT^29,36^ (see Supplementary Note 6 for a summary of the method and setup). Figure 2 shows the subsequently applied ^13^C magnetometry protocol at 7 T. It entails a train of *θ* pulses, spin-locking the nuclear spins along $\hat{{{{{{{{\bf{x}}}}}}}}}$ (Fig. 2A). ^13^C nuclei remain in quasi-equilibrium along $\hat{{{{{{{{\bf{x}}}}}}}}}$ for several seconds^31^. Flip angle *θ* can be arbitrarily chosen, except for *θ* = *π*^34^. Pulse duty cycle is high (19–54%) and interpulse spacing *τ* < 100 *μ*s (Fig. 2A). The nuclei are inductively interrogated in *t*_acq_ windows between pulses. Orange curves in Fig. 2B show typical raw data. For each window, the magnitude of the heterodyned Larmor precession, (Fig. 2C, here at 20 MHz)—effectively the rotating frame transverse magnetization component—constitutes the magnetometer signal. These are the blue points in Fig. 2D. Every pulse, therefore, provides one such point; in typical experiments we apply *N* ≳ 200k pulses. In the absence of an external field, successive points show very little decay. We typically achieve transverse spin lifetimes exceeding >30 s; remarkably however, this is accomplished using a pulsing rate that is only a factor of 10-fold faster than the interspin dipolar coupling. Now, when subject to an AC field at *f*_AC_, there is an oscillatory response (evident in blue line Fig. 2D). It is strongest at the "resonance condition”,

1 $${f}_{{{{{{{{\rm{res}}}}}}}}}=\frac{\theta }{2\pi \tau },$$

as will be explained below, corresponding to when the AC field periodicity is matched to the time to complete a 2*π* rotation. Figure 2A describes the resonant situation for *θ* ≈ *π*/2.

**Fig. 3:**
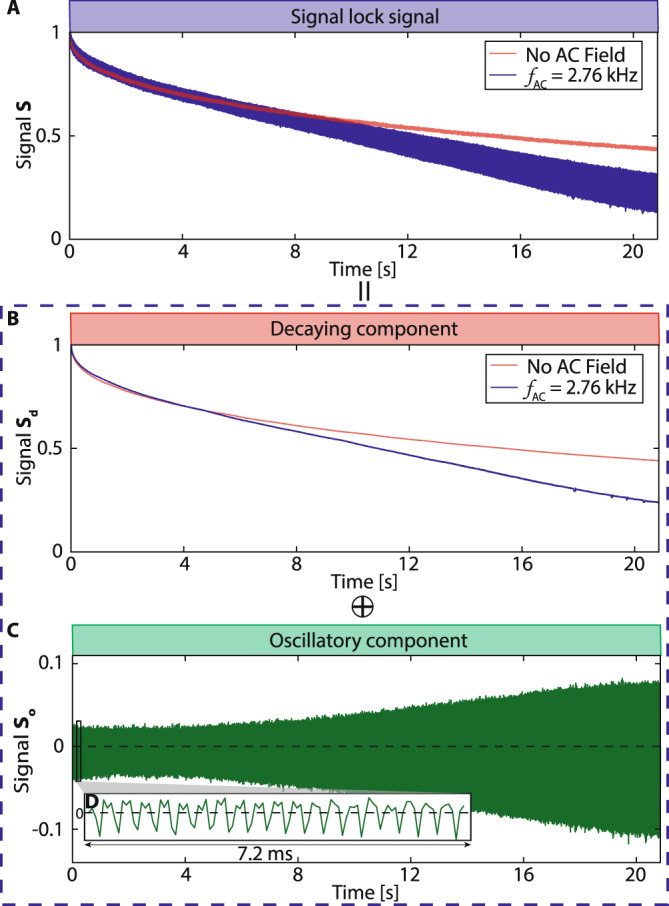
Long-time ^13^C magnetometry signal. **A** Representative pulsed spin-lock signal as in Fig. 2 (*τ* = 73*μs*, *t*_*p*_ = 30 *μ*s) with no applied field (red line), and with *f*_AC_ = 2.76 kHz (blue line), applied near resonance (${f}_{{{{{{{{\rm{res}}}}}}}}}=2.780$ kHz). The former decays with ${T}_{2}^{\prime}=$31 s; AC field, however, yields a faster decay with superimposed oscillations (see Fig. 2A). **B** Decaying signal component *S*_*d*_ obtained via a moving average filter. **C** Oscillatory signal component *S*_*o*_ extracted as *S*_*o*_ = *S* − *S*_*d*_. **D** Inset: zoom into data in a 7.2 ms window (boxed) showing oscillations at *f*_AC_ (see Fig. 5).

Figure 3 shows the magnetometer signal over 20 s (275k pulses) with no applied field and *f*_AC_ = 2.760 kHz (close to resonance). In the former case, there is a slow decay ${T}_{2}^{\prime}=31$ s (red line in Fig. 3A)^31^. The AC field however drives the spins away from $\hat{{{{{{{{\bf{x}}}}}}}}}$, causing a comparatively rapid ^13^C decay (blue line in Fig. 3A) along with magnetization oscillations (see Fig. 3C). We separate these contributions, decomposing the signal (dashed box) as *S* = *S*_*d*_ + *S*_*o*_, where *S*_*d*_ is the (slow) decay component (Fig. 3B) and *S*_*o*_ is the oscillatory part with zero mean (Fig. 3C, zoomed in Fig. 3D). In practice, *S*_*d*_ is obtained via a 73 ms moving average filter applied to *S* in Fig. 2, and the oscillatory component isolated as *S*_*o*_ = *S* − *S*_*d*_.

**Fig. 4:**
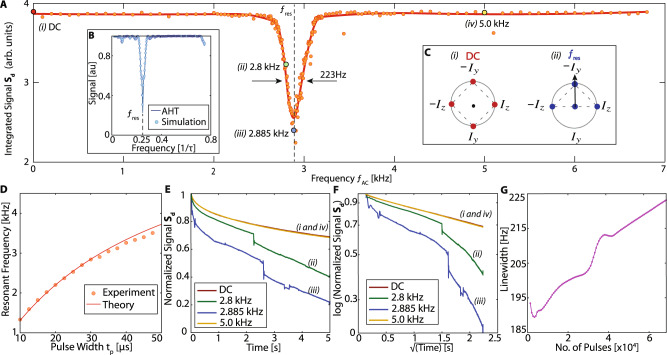
Enhanced signal decay with applied AC field. **A** Integrated signal intensity of decaying component *S*_*d*_ (see Fig. 3B) with changing AC frequency, with *θ* ≈ 75^∘^, and *τ* = 73 μs. Decays are normalized against their value at 20 ms and truncated at 5 s (corresponding to ≈ 10^5^ pulses). Enhanced decay occurs at resonance condition (dashed line). Solid line is a spline fit guide to the eye. Linewidth is estimated ≈ 223 Hz (marked) from a Gaussian fit. **B** Average Hamiltonian analysis. Simulated signal, here assuming *θ* = *π*/2 and no dipolar coupling between ^13^C nuclei. Resonance is expected at ${f}_{{{{{{{{\rm{res}}}}}}}}}=1/4\tau$. **C** Phasor representation of toggling frame Hamiltonians for **B** under an applied (i) DC field and (ii) AC field at resonance. **D** Resonance frequency scaling. Points show experimentally extracted resonance frequency from a second harmonic intensity (see Fig. S1) for varying pulse widths *t*_*p*_ but fixed *t*_acq_. Solid line is a theoretically predicted ${f}_{{{{{{{{\rm{res}}}}}}}}}$, showing good agreement. **E** Signal traces for representative points in **A** (large markers i-iv), corresponding to *f*_AC_ = DC, 5 kHz, 2.885 kHz (resonance) and 2.8 kHz (slightly off-resonance). For AC fields near resonance (ii,iii), we observe rapid decays and sharp jumps (see See supplementary online material). Far from resonance (i,iv), the decays exhibit slowly-decaying profiles. **F** Logarithmic scale plot of the data in **E**, plotted against $\sqrt{t}$. Signal decays far from the resonance have a characteristic $\propto \exp (-{t}^{1/2})$ profile^31^. Decays close to resonance (ii,iii), however, show steeper slopes. **G** Scaling of resonance linewidth in **A** as a function of the number of pulses *N* applied, estimated from a Gaussian fit.

We first focus attention to the decay component *S*_*d*_. In Fig. 4, we vary *f*_AC_, and study the change in integrated value of *S*_*d*_ over a 5 s period (Fig. 3B). Here the phase of the applied AC field is random. Figure 4A reveals that the ^13^C decay is unaffected for a wide range of frequencies, except for a sharp decay response (dip) centered at resonance ${f}_{{{{{{{{\rm{res}}}}}}}}}$ (dashed vertical line). With a Gaussian fit, we estimate a linewidth ≈ 223 Hz. The dip matches intuition developed from average Hamiltonian theory (AHT) (Fig. 4B, C) (see below). It can be considered to be an extension of dynamical decoupling (DD) sensing^41,42^ for arbitrary *θ*. Figure 4D shows the scaling of ${f}_{{{{{{{{\rm{res}}}}}}}}}$ with pulse width *t*_*p*_. This agrees well with theoretical scaling, ${f}_{{{{{{{{\rm{res}}}}}}}}}=A{t}_{p}/({t}_{p}+{t}_{{{{{{{{\rm{dead}}}}}}}}}+{t}_{{{{{{{{\rm{acq}}}}}}}}})$, where *A* is a measure of the Rabi frequency.

Figure 4E, F, display individual decays in Fig. 4A on a linear scale and logarithmic scale against $\sqrt{t}$. Points far from resonance (e.g., (i) DC and (iv) 5 kHz) exhibit a stretched exponential decay $\propto \exp (-{t}^{1/2})$, characteristic of interactions with the P1 center spin bath^31^. These manifest as the straight lines in Fig. 4F. On the other hand, for points within the ${f}_{{{{{{{{\rm{res}}}}}}}}}$ dip in Fig. 4A, we observe a potentially chaotic response evidenced by sharp jumps (Fig. 4E, F). These features belie a simple explanation from AHT alone and is possibly an artifact from our probe like arcing. The jumps occur for strong Rabi frequencies, and when *f*_AC_ approaches resonance, evident in the comparison between *f*_AC_ = 5kHz and *f*_AC_ = 2.885 kHz in Fig. 4E, F (blue and yellow lines). Figure 4G describes the linewidth dependence of the obtained resonance dip as a function of the number of pulses employed. Contrary to DD sensing^10,43–45^, the linewidth does not fall with increasing number of pulses, suggesting it is dominated by ^13^C dipolar couplings.

**Fig. 5:**
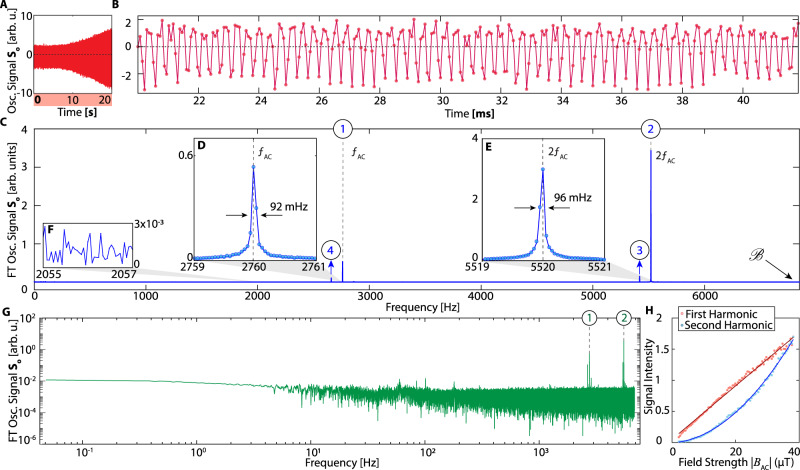
High-field (7T) ^13^C sensor magnetometry. **A** Long time single-shot signal of the oscillatory component *S*_*o*_ (following Fig. 3C) upon application of a *f*_AC_ = 2.760 kHz AC field. Here *τ* = 73*μ*s, *θ* ≈ 75^∘^, and ${f}_{{{{{{{{\rm{res}}}}}}}}}\,=2.78$ kHz. **B** Zoom into a 20 ms window. **C** Fourier transform of data in **A** reveals high resolution peaks corresponding harmonics of *f*_AC_. Data here is obtained from 100 averages of signal as in **A**. Dashed lines represent first and second harmonics. Third and fourth harmonics are aliased, bandwidth ${{{{{{{\mathcal{B}}}}}}}}=6849$ Hz. **D**, **E** Zoom into primary and secondary harmonics centered at *f*_AC_ and 2*f*_AC_ respectively. Linewidths are estimated from a Gaussian fit. **F** Zoom into spectral wing showing noise intensity. **G** Logarithmic plot corresponding to data in **C**. **H** Scaling of harmonic intensity. Primary and secondary harmonic intensities exhibit apparently linear and quadratic dependence with ∣*B*_AC_∣ respectively for an off-resonant AC field at 1.75 kHz and with a different probe power such that *τ* = 120 *μs*.

Despite this relatively broad linewidth, high resolution magnetometry can be extracted from the oscillatory component *S*_*o*_ (Fig. 3C). Figure 5A shows *S*_*o*_ over 20 s for *f*_AC_ = 2.760 kHz. Figure 5B zooms into a representative 22 ms window. Strong ^13^C oscillations are evident here. Taking a Fourier transform, we observe four sharp peaks (Fig. 5C). We identify the two strongest peaks as being exactly at *f*_AC_ and 2*f*_AC_ (shown in Fig. 5C with the labels ① and ②); we will refer to them as primary and secondary harmonics respectively. They are zoomed for clarity in Fig. 5D, E (along with the noise level in Fig. 5F), from which we extract the AC magnetometry linewidths as 92 mHz and 96 mHz respectively. Two other smaller peaks in Fig. 5C are aliased versions of the third and fourth harmonics (marked ③ − ④), at frequencies ${f}_{3}=2{{{{{{{\mathcal{B}}}}}}}}-3{f}_{{{{{{{{\rm{AC}}}}}}}}}=5.418$ kHz and ${f}_{4}=2{{{{{{{\mathcal{B}}}}}}}}-4{f}_{{{{{{{{\rm{AC}}}}}}}}}=2.658$ kHz. The bandwidth ${{{{{{{\mathcal{B}}}}}}}}=1/(2\tau )$ here is determined by the interpulse interval in Fig. 2A. For clarity, Fig. 5G shows the data in Fig. 5C in a logarithmic scale, with the harmonics marked.

We emphasize differences with respect to DD quantum sensing^10,11^. Oscillations here are at the absolute AC frequency *f*_AC_, as opposed to the difference frequency from *τ*^−1^ in the DD case. Second, sensing can be obtained with an arbitrary flip angle *θ* (except *π*), allowing for greater robustness compared to DD sequences that require precisely calibrated *θ* = *π* pulses. Pulse error affects AC magnetometry peak intensity but not their position. Figure 5H shows the scaling of the harmonic intensities with ∣*B*_AC_∣. We observe a linear and quadratic dependence of the primary and secondary harmonic intensities (see Fig. 5H), differing from DD sensing.

**Fig. 6:**
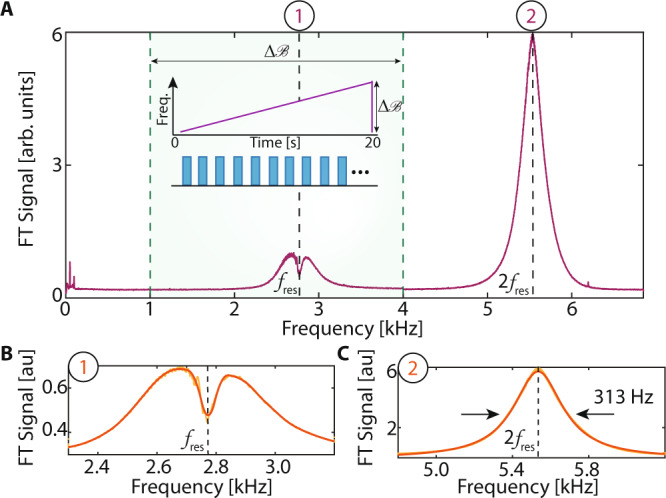
Frequency response of ^13^C magnetometer. **A** Spectral response of an chirped AC field (inset) where frequency is swept between 1 and 4 kHz ($\Delta {{{{{{{\mathcal{B}}}}}}}}=3$kHz), (green shaded region) in 20 s. Plotted is the resulting Fourier transform intensity of *S*_*o*_ (averaged over 100 shots) revealing profile of sensor frequency response. Data is smoothed over 10 Hz for clarity. Primary and secondary harmonic responses are marked. Dashed lines denote ${f}_{{{{{{{{\rm{res}}}}}}}}}$ and $2{f}_{{{{{{{{\rm{res}}}}}}}}}$. **B** Zoom into primary harmonic response. Points show cusp-like primary harmonic response with a linewidth of ≈ 60 Hz about ${f}_{{{{{{{{\rm{res}}}}}}}}}=2.78$ kHz (dashed line). Orange solid line is a spline fit guide to the eye. **C** Zoom into secondary harmonic response showing an approximately Gaussian profile, here with a linewidth of ≈ 313 Hz, centered at 2${f}_{{{{{{{{\rm{res}}}}}}}}}$.

We now perform experiments to determine the frequency response of the sensor, unraveling the sensitivity profile at different frequencies (Fig. 6). We seek to determine how it relates to the ${f}_{{{{{{{{\rm{res}}}}}}}}}$ dip in Fig. 4. We apply a chirped AC field, ${f}_{{{{{{{{\rm{AC}}}}}}}}}(t)={f}_{{{{{{{{\rm{ini}}}}}}}}}+(\Delta {{{{{{{\mathcal{B}}}}}}}}/T)t$, with *f*_ini_ = 1 kHz, in a 1–4 kHz window ($\Delta {{{{{{{\mathcal{B}}}}}}}}=3$kHz) in Fig. 6A (shaded region. The sweep is slow, (*T* = 20 s), and occurs only once during the full sequence, and does not start synchronously with it. If the frequency response was independent of frequency, we would expect an approximately box-like Fourier signal intensity over $\Delta {{{{{{{\mathcal{B}}}}}}}}$. Instead, we obtain a narrow response with a central cusp in a small portion of the $\Delta {{{{{{{\mathcal{B}}}}}}}}$ band (zoomed in Fig. 6B). This response is strongest close to resonance (Eq. (1)), a signature of the sign inversion of the ^13^C spins due to the rapid adiabatic passage driven by the AC field in the rotating frame. Upon hitting the resonance condition, the spins tip towards the $\hat{{{{{{{{\bf{y}}}}}}}}}-\hat{{{{{{{{\bf{z}}}}}}}}}$ plane and their amplitude in the $\hat{{{{{{{{\bf{x}}}}}}}}}-\hat{{{{{{{{\bf{y}}}}}}}}}$ plane, as measured, reduces. Simultaneously, we observe a strong Gaussian response in the region outside $\Delta {{{{{{{\mathcal{B}}}}}}}}$ (Fig. 6C). We identify these features as the primary and secondary harmonic responses, respectively (labeled ① and ② as before). Indeed, their frequency centroids are in the ratio 1:2 (see Fig. 5C); ${f}_{{{{{{{{\rm{res}}}}}}}}}$ arises where the cusp dips to its lowest point (dashed line in Fig. 6B). We hypothesize that the cusp arises because the actual signal response reflects a dispersive lineshape, flipping in sign on either side of resonance ${f}_{{{{{{{{\rm{res}}}}}}}}}$ (see Sec. II B).

**Fig. 7:**
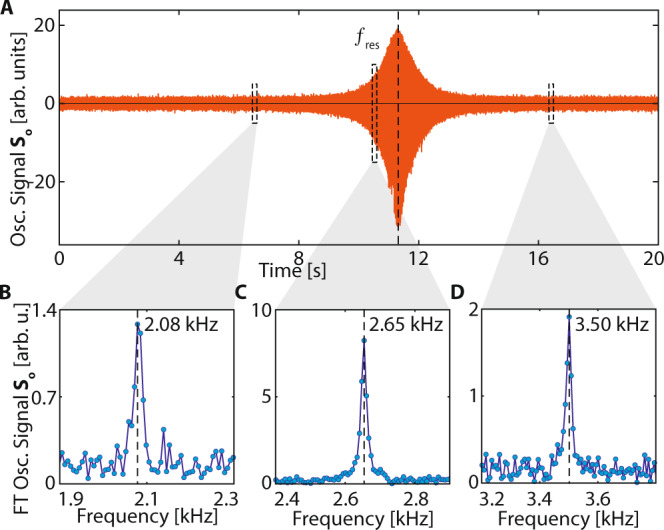
Tracking signals in time domain at 7T using a ^13^C magnetometer. **A** Long-time oscillatory response *S*_*o*_ under the chirped AC field in Fig. 6A. Signal shows a strong response as the instantaneous frequency cross resonance ${f}_{{{{{{{{\rm{res}}}}}}}}}$. **B**–**D** Tracking of AC frequency. Three 150 ms time windows are marked, and panels **B**–**D** show the corresponding Fourier transforms. Solid lines are Lorentzian fits. Clearly the ^13^C signal carries a real-time imprint of the frequency of the applied chirped AC magnetic field. A more detailed and complete account of this analysis is given in Fig. S8 including a short-time Fourier transform.

Figure 6 suggests the potential for real-time magnetic field "tracking”. We consider this experimentally in Fig. 7. Figure 7A shows *S*_*o*_ corresponding to a single-shot of the experiment in Fig. 6A. Sensor response is enhanced as the frequency crosses resonance ${f}_{{{{{{{{\rm{res}}}}}}}}}$ (dashed line). Three 15 ms windows are displayed in Fig. 7B. Their Fourier transforms reveal that the frequency response follows the instantaneous AC field *f*_AC_(*t*).

### Theory

We now turn to a theoretical description of ^13^C sensor operation. We outline two complementary viewpoints to explain the observations in Figs. 4 and 5: a first picture equivalent to DD quantum sensing, and second using an "rotating-frame” NMR experiment analogue. Consider first that the ^13^C Hamiltonian in the lab-frame is, ${{{{{{{\mathcal{H}}}}}}}}={{{{{{{{\mathcal{H}}}}}}}}}_{{{{{{{{\rm{Z}}}}}}}}}+{{{{{{{{\mathcal{H}}}}}}}}}_{{{{{{{{\rm{dd}}}}}}}}}+{{{{{{{{\mathcal{H}}}}}}}}}_{{{{{{{{\rm{AC}}}}}}}}}$, where ${{{{{{{{\mathcal{H}}}}}}}}}_{{{{{{{{\rm{Z}}}}}}}}}={w}_{{{{{{{{\rm{L}}}}}}}}}{I}_{z}$ is the Zeeman Hamiltonian, ${{{{{{{{\mathcal{H}}}}}}}}}_{{{{{{{{\rm{dd}}}}}}}}}={\sum }_{k\,{ < }\,\ell }{b}_{k\ell }(3{I}_{kz}{I}_{\ell z}-\overrightarrow{{I}_{k}}\cdot \overrightarrow{{I}_{\ell }})$ is the interspin dipolar interaction and ${{{{{{{{\mathcal{H}}}}}}}}}_{{{{{{{{\rm{AC}}}}}}}}}={\gamma }_{n}{B}_{{{{{{{{\rm{AC}}}}}}}}}\cos (2\pi {f}_{{{{{{{{\rm{AC}}}}}}}}}t+{\varphi }_{0}){I}_{z}$ is the applied AC field. Here *I* refer to spin-1/2 Pauli matrices, *w*_L_ is the nuclear Larmor frequency, *φ*_0_ is the initial (arbitrary) phase of the AC field, and we estimate the median dipolar coupling $J=\left\langle {b}_{k\ell }\right\rangle \, \approx$ 663Hz. The spins are prepared initially along $\hat{{{{{{{{\bf{x}}}}}}}}}$ in a state *ρ*(0) ~ *εI*_*x*_, where *ε* ≈ 0.2% is the hyperpolarization level.

The sequence in Fig. 2A can be conveniently treated in the rotating frame by average Hamiltonian theory (AHT)^46^. After *N* pulses, its action can be described by the unitary, $U(N\tau )={\left[\exp (i\theta {I}_{x})\exp (i{{{{{{{\mathcal{H}}}}}}}}\tau )\right]}^{N}$, where we assume *δ*-pulses for simplicity. The signal obtained following the procedure in Fig. 2 then simply corresponds to the measurement of ${[{\langle {I}_{x}\rangle }^{2}+{\langle {I}_{y}\rangle }^{2}]}^{1/2}$, or equivalently the spin survival probability in the $\hat{{{{{{{{\bf{x}}}}}}}}}-\hat{{{{{{{{\bf{y}}}}}}}}}$ plane. This evolution can be expressed as, $U(N\tau )=\mathop{\prod }\nolimits_{j=1}^{N}\exp (i{{{{{{{{\mathcal{H}}}}}}}}}^{(\,\,j)}\tau )$, where ${{{{{{{{\mathcal{H}}}}}}}}}^{(j)}$ are toggling frame Hamiltonians after every pulse^46^, ${{{{{{{{\mathcal{H}}}}}}}}}^{(j)}=\exp (ij\theta {I}_{x}){{{{{{{\mathcal{H}}}}}}}}\exp (-ij\theta {I}_{x})$. For time *t* = *Nτ*, this can be recast as, $U(t)=\exp (i{{{{{{{{\mathcal{H}}}}}}}}}_{F}N\tau )$, where ${{{{{{{{\mathcal{H}}}}}}}}}_{F}$ captures the effective system dynamics under the pulses. To leading order in parameter *ζ* = 2*πJτ* in a Magnus expansion^47–49^, and assuming *ζ* ≪ 1, the dynamics can be captured by an average Hamiltonian, ${{{{{{{{\mathcal{H}}}}}}}}}_{F}^{(0)}=\mathop{\sum }\nolimits_{j=1}^{N}{{{{{{{{\mathcal{H}}}}}}}}}^{(j)}$.

Since non-commuting effects do not affect the leading order term, we can consider the effect of each of the dipolar part and the AC field separately, ${{{{{{{{\mathcal{H}}}}}}}}}_{F}^{(0)}={{{{{{{{\mathcal{H}}}}}}}}}_{{{{{{{{\rm{dd}}}}}}}}}^{(0)}+{{{{{{{{\mathcal{H}}}}}}}}}_{{{{{{{{\rm{AC}}}}}}}}}^{(0)}$. For the former, we have^34^,

2 $${{{{{{{{\mathcal{H}}}}}}}}}_{{{{{{{{\rm{dd}}}}}}}}}^{(0)}=\mathop{\sum }\limits_{j=1}^{N}{{{{{{{{\mathcal{H}}}}}}}}}_{{{{{{{{\rm{dd}}}}}}}}}^{(j)}\approx \mathop{\sum}\limits_{j < k}{d}_{jk}^{{{{{{{{\rm{CC}}}}}}}}}\left(\frac{3}{2}{{{{{{{{\mathcal{H}}}}}}}}}_{{{{{{{{\rm{ff}}}}}}}}}-\overrightarrow{{I}_{j}}\cdot \overrightarrow{{I}_{k}}\right),$$

with the flip-flop Hamiltonian, ${{{{{{{{\mathcal{H}}}}}}}}}_{{{{{{{{\rm{ff}}}}}}}}}={I}_{jz}{I}_{kz}+{I}_{jy}{I}_{ky}$. The initial state *ρ*(0) is conserved under ${{{{{{{{\mathcal{H}}}}}}}}}_{{{{{{{{\rm{dd}}}}}}}}}^{(0)}$, since $[\rho (0),{{{{{{{{\mathcal{H}}}}}}}}}_{{{{{{{{\rm{dd}}}}}}}}}^{(0)}]=0$. This leads to a quasi-equilibriation of spins along $\hat{{{{{{{{\bf{x}}}}}}}}}$, with a lifetime that scales with a power law of the pulsing frequency *τ*^−1^^31^. This is the red signal in Fig. 3A (here *ζ* = 0.3). We note that for sufficiently small *ζ*, Eq. (2) is valid for arbitrary flip-angle *θ*, except for certain special values (*θ* ≈ *π*, 2*π*).

A similar AHT analysis can be carried out for the ${{{{{{{{\mathcal{H}}}}}}}}}_{{{{{{{{\rm{AC}}}}}}}}}$ term. Consider first a DC field (*f*_AC_ = 0) and *θ* = *π*/2. Figure 2C shows the toggling frame Hamiltonians ${{{{{{{{\mathcal{H}}}}}}}}}_{{{{{{{{\rm{AC}}}}}}}}}^{(\,\,j)}$, which consists only of single body terms and hence can be plotted in a phasor representation. In a cycle consisting of four pulses (required to complete a 2*π* rotation), the average Hamiltonian ${{{{{{{{\mathcal{H}}}}}}}}}_{{{{{{{{\rm{AC}}}}}}}}}^{(0)}=0$, evident from the symmetrically distributed phasors in Fig. 4(i). Hence the DC field is decoupled. Alternately, consider the resonant AC case (${f}_{{{{{{{{\rm{AC}}}}}}}}}={f}_{{{{{{{{\rm{res}}}}}}}}}$). The analysis here is simplest to carry out assuming a square-wave (as opposed to sinusoidal) field. In this situation, the phasor diagram is asymmetrical and the average Hamiltonian after four pulses, ${{{{{{{{\mathcal{H}}}}}}}}}_{{{{{{{{\rm{AC}}}}}}}}}^{(0)}\propto -{I}_{y}$. When stroboscopically observed, the spins rotate away from $\hat{{{{{{{{\bf{x}}}}}}}}}-\hat{{{{{{{{\bf{y}}}}}}}}}$ plane; this yields the dip in the integrated signal in Fig. 4.

An illuminating alternate viewpoint is obtained by noting that under spin-locking the spins are requantized in the rotating frame with an effective field, ${\Omega }_{{{{{{{{\rm{eff}}}}}}}}}=\Omega \left(\frac{{t}_{p}}{\tau }\right)$, where the factor in brackets is the pulsing duty cycle, and Ω is the Rabi frequency. The resonance frequency identified above is then exactly, ${f}_{{{{{{{{\rm{res}}}}}}}}}={\Omega }_{{{{{{{{\rm{eff}}}}}}}}}$. Therefore the experiment can cast as an rotating-frame analogue of a conventional (lab-frame) NMR experiment: the spins are quantized along $\hat{{{{{{{{\bf{x}}}}}}}}}$, Ω_eff_ serves analogous to the Larmor frequency, and at the resonance condition (${f}_{{{{{{{{\rm{AC}}}}}}}}}={f}_{{{{{{{{\rm{res}}}}}}}}}$), *B*_AC_ is the effective Rabi frequency. Spins initially prepared along $\hat{{{{{{{{\bf{x}}}}}}}}}$, are then constantly tipped away from this axis by *f*_AC_. The dip in Fig. 4 reflects this tilt away from the $\hat{{{{{{{{\bf{x}}}}}}}}}-\hat{{{{{{{{\bf{y}}}}}}}}}$ plane. For the resonant case, in the absence of dipolar evolution, the trajectory of the spins in the rotating frame can be simply written as,

3 $$\rho (t)={I}_{x}\cos ({\gamma }_{n}{B}_{{{{{{{{\rm{AC}}}}}}}}}t)\,	+{I}_{y}\sin ({\gamma }_{n}{B}_{{{{{{{{\rm{AC}}}}}}}}}t)\sin (2\pi {f}_{{{{{{{{\rm{AC}}}}}}}}}t)\\ \,	+{I}_{z}\sin ({\gamma }_{n}{B}_{{{{{{{{\rm{AC}}}}}}}}}t)\cos (2\pi {f}_{{{{{{{{\rm{AC}}}}}}}}}t).$$

In effect, the spins are undergoing a "secondary” precession in the rotating frame around $\hat{{{{{{{{\bf{x}}}}}}}}}$ at frequency Ω_eff_. For each point on this motion, they are also precessing in the lab frame at *w*_L_. The latter yields the inductive signal measured in the raw data in Fig. 2B. Upon taking a Fourier transform (Fig. 2C), we are able to extract the magnitude of the spin vector in the $\hat{{{{{{{{\bf{x}}}}}}}}}-\hat{{{{{{{{\bf{y}}}}}}}}}$ plane, which has the form, $S(t)={[{\cos }^{2}({\gamma }_{n}{B}_{{{{{{{{\rm{AC}}}}}}}}}t)+{\sin }^{2}({\gamma }_{n}{B}_{{{{{{{{\rm{AC}}}}}}}}}t){\sin }^{2}(2\pi {f}_{{{{{{{{\rm{AC}}}}}}}}}t)]}^{1/2}$. This is the signal measured in Fig. 3, and the oscillations here at *f*_AC_ and 2*f*_AC_, corresponding to the observed first and second harmonics respectively. While this analysis was for the resonant case, it is simple to extend it to off-resonant AC fields (see Supplementary Note 9). Once again the oscillations can be demonstrated to be at exact harmonics of *f*_AC_. Alternatively, if the FT phase, instead of magnitude (see Supplementary Note 5), was taken in Fig. 2C, where one measures the phase of the spins in the $\hat{{{{{{{{\bf{x}}}}}}}}}-\hat{{{{{{{{\bf{y}}}}}}}}}$ plane in the rotating frame, Eq. (3) indicates there will once again be oscillations. Here however, all the intensity will be in the primary harmonic, and for sufficiently small fields *γ*_*n*_∣*B*_AC_∣ ≪ *f*_AC_, the oscillatory signal scales ∝ ∣*B*_AC_∣, and can provide higher sensitivity. Extracting these oscillations comes with complications related to unwrapping the phase every 2*π*, and accounting for phase accrual during the *t*_*p*_ pulse periods. Supplementary Note 5 elucidates the phase unwrapping strategy employed in this work.

Overall Eq. (3) illustrates that the oscillations in Fig. 5 are akin to observing the Larmor precession of the spins in the rotating frame. The growing oscillation strength in Fig. 5A might indicate the spins tipping further away from the $\hat{{{{{{{{\bf{x}}}}}}}}}$ axis. In a similar manner, Eq. (3) demonstrates that Figs. 6 and 7 are analogous to the AC field driving a rapid adiabatic passage in the rotating frame. Therefore, the sign of $\left\langle {I}_{x}\right\rangle$ flips on either side of the resonance (see Fig. 6).

Finally, we emphasize that the treatment above is still only qualitative because it neglects the effect of the dipolar interactions during the full spin-locking sequence. An interplay between the dipolar interaction and AC field is ultimately important in determining the transverse lifetimes upon spin excursions away from the $\hat{{{{{{{{\bf{x}}}}}}}}}$ axis. We will consider this in detail in follow-up work.

### ^13^C sensor performance and special features

We now elucidate parameters of ^13^C sensor performance and discuss some of its special features (see SI (See supplementary online material) for a summary). Our emphasis in this paper was not to optimize sensitivity, but through experiments similar to Fig. 4 (detailed in Supplementary Note 11) for a bias field *B*_0_ = 7 T and at the resonance frequency, we obtain a single-shot sensitivity of 760 ± 127 pT$/\sqrt{{{{{{{{\rm{Hz}}}}}}}}}$ via measurement of signal amplitude, and 410 ± 90 pT$/\sqrt{{{{{{{{\rm{Hz}}}}}}}}}$ via measurement of signal phase respectively. This neglects hyperpolarization time because a sensor initialization event can allow interrogation for several minutes^31^. A single-shot of the measurement (for *t* = 34s at ${f}_{{{{{{{{\rm{res}}}}}}}}}$) can detect a minimum field of ≈ 70 pT. While the sensitivity here is lower than NV quantum sensors at low field, we emphasize that this corresponds to an exquisite AC field precision of ~ 10^−11^ over the 7 T bias field. Indeed, as elucidated in Supplementary Note 13, our ^13^C sensor occupies a niche space of high-field operation with few competing technologies (MOKE, SHPM, and ^1^H NMR). While possessing comparable sensitivity, it expands applications to multiplexed sensing of AC fields that is not easily feasible via these sensor technologies.

Sensitivity itself can be significantly enhanced. Sample filling factor (presently *η* ≈ 15%) and RF coil Q-factor ( ≈ 30) can both be significantly boosted. Employing a 10% ^13^C enriched sample would provide a further ten-fold gain in inductive signal strength^34^. Current hyperpolarization levels are around ( ≈ 0.1%), but we anticipate that technical improvements can boost this by a further 50-fold^50^. Experiments in this paper have been conducted on a single crystal sample, but we anticipate similar long coherence times ${T}_{2}^{\prime}$ can be accomplished for nanodiamond (ND) samples via methods of high-temperature sample thermal annealing^51^. From these concerted gains, we estimate a sensitivity approaching 2 nT$/\sqrt{\,{{\mbox{Hz}}}}$ is feasible for ^13^C-enabled NMR detection in a (10 μm)^3^ volume, sufficient to measure the ~ 11 nT field produced by precessing ^1^H nuclei of glycerol in the same volume at 10 T^12^.

The frequency resolution of the ^13^C sensor is *δf* ≈ 1/*Nτ*. Currently, Fig. 5 demonstrates a resolution ~ 50 mHz. However, finite memory limitations restricted capturing the ^13^C Larmor precession here to *t* < 35s (Fig. 5A). Overcoming these memory limits can allow acquisition of the entire spin-lock decay, lasting over 573 s^31^. Under these conditions, we estimate a frequency resolution of 2.2 mHz is feasible. This would correspond to a frequency precision of 3 ppt at a 7 T bias field. On the other hand, sensor bandwidth ${{{{{{{\mathcal{B}}}}}}}}=1/2\tau$ is determined by the minimum interpulse delay (see Fig. 4C). Current Rabi frequencies limit bandwidth to ~ 20 kHz. We estimate that improving filling-factor *η* and RF coil Q factor (presently 30), could increase ${{{{{{{\mathcal{B}}}}}}}}$ further to ≈ 500 kHz. The sensor strategy lends itself to a wide operating field range. While our experiments were carried out at 7 T, the slow scaling ^13^C gyromagnetic ratio makes sensing viable even for fields ≳ 24 T. This greatly expands the field range for spin sensors, where the operating field is predominantly < 0.3 T (notable exceptions are Refs. 13,15, but require complex instrumentation).

We emphasize the robustness of our sensing method to pulse error. It can be operated with any flip angle *θ* ≠ *π*^34^. Moreover, ${f}_{{{{{{{{\rm{res}}}}}}}}}$ has a relatively wide profile ≈ 230 Hz (see Fig. 4A), meaning that flip-angle (RF) inhomogeneity has little impact. These features are responsible for the > 275k pulses applied to the ^13^C spins in experiments in this work.

Finally, we note some special features of our magnetometry protocol. Compared to previous work^52^ using hyperpolarized gaseous ^129^Xe nuclei as sensors, our experiments employed hyperpolarized nuclear spins in solids. This provides natural advantages due to an ability for in-situ replenishment of hyperpolarization at the sensing site. Moreover, multiple AC fields can be discerned in a Fourier reconstruction in a single-shot, as opposed to point-by-point^52^.

Fundamentally, experiments here illustrate the feasibility of quantum sensing in the coupled sensor limit ($< d > {T}_{2}^{*} \sim 1$)^53,54^, making the spins sensitive to external fields while negating the effect of intersensor interactions. Sensor operation exploits "Floquet prethermalization”^55,56^—quasi-equilibrium nuclear states under periodic driving^31^. As such, this provides a compelling demonstration of exploiting stable non-equilibrium phases for sensing applications.

From a technological perspective, RF interrogated sensing, as described here, presents advantages in scattering environments^37^ (see Supplementary Note 12). All data in this paper are carried out with the diamond immersed in ~ 4mL water, over 2000-fold the volume of the sample. Traditional NV sensors are ineffective in this regime due to scattering losses and concomitant fluorescence fluctuations. Similarly, optically hyperpolarized sensors present advantages because majority of the sensor volume can be illuminated by the impinging lasers, because there are no geometrical constraints from the requirements of collection optics. In our experiments, we employed an array of low-cost laser diode sources^50^ for hyperpolarization that illuminates the sample almost isotropically. This allows recruiting a larger volume of spins for sensing with a low cost overhead^50^. Extension to powder samples could be advantageous for optimally packing a sensor volume.

## Discussion

The work presented here can be extended in several promising directions. First, the high bandwidth-to-resolution ratio (${{{{{{{\mathcal{B}}}}}}}}/\delta f\,\approx\, 1{0}^{5}$) possible via ^13^C sensing at high fields (see Fig. 5), suggests possibilities for detecting chemical shifts from processing analyte nuclei external to the diamond. Seminal work by Warren et al.^57^ and Bowtell^58^ showed that nuclear spins of one species ("sensor”) could be used to indirectly probe NMR information of other physically separated ("analyte”) nuclei. This indirect NMR detection strategy is appealing as the absolute dimensions diminish to sub-micron length scales^2^. Building on these ideas, we envision the possibility of micro-scale NMR detectors using hyperpolarized ^13^C nuclei in nanodiamond (ND) particles, and exposing one-half of the particle surfaces to the analyte^57^. Such "relayed" NMR detection is feasible because of the high resolution-to-bandwidth ratio of our ^13^C magnetometer ( < 10 ppm). For instance, the detection of ^1^H analyte chemical shifts would require the translation of the corresponding nuclear Larmor precession into the magnetometer detection bandwidth ( ~ 7 kHz)^59,60^. Such frequency translation is possible following the approach first introduced by Wittfield and Redfield^61^ of detecting oscillating longitudinal spin magnetization at audio-frequencies. In principle, this requires additional control applied to the analyte nuclei. For instance, a train of *π*/2 pulses applied to the ^1^H nuclei coincident with the spin-locking train applied to the ^13^C sensor nuclei in Fig. 2A allows ^1^H chemical shift information to be encoded into longitudinal ^1^H magnetization oscillations and hence still retained. Ultimately, the spatial distance over which the sensing extends will be determined approximately by the size of the ^13^C sensor ensemble employed^12^, hence opening the possibility of micron-scale NMR detection. We will explore these experiments in a future manuscript.

Second, while the current experiments exploit a low-field DNP mechanism for the initialization of the ^13^C sensors, interesting opportunities arise from employing complementary all-optical DNP techniques that operates directly at high field^62,63^. This will allow in-situ sensors at high field without the need for sample shuttling.

Furthermore, beyond NMR, we envision applications to condensed matter physics. Our approach might enable non-invasive and high spatial resolution visualization of the observed phenomenology of domain walls in 2D ferromagnets^22,23^, elucidation of coexisting antiferromagnetic and spin-glass phases in 2D crystals^25^, and reentrant Hofstadter phases in twisted bilayer graphene at high magnetic fields^27^.

More broadly, this work suggests an interesting applications of DNP for quantum sensing. It is intriguing to consider sensor platforms constructed in optically-active molecular systems^64^ where abundant, long-lived nuclear spins, can be initialized via interactions with electronic spin centers. Finally, we envision technological applications of the ^13^C sensors described here for bulk magnetometry^65^, sensors underwater and in scattering media, and for spin gyroscopes^66–69^.

In conclusion, we have proposed and demonstrated a high-field magnetometry approach with hyperpolarized ^13^C nuclear spins in diamond. Sensing leveraged long transverse spin ^13^C lifetimes and their ability to be continuously interrogated, while mitigating effects due to interspin interaction. We demonstrated magnetometry with high frequency resolution ( ~ 50 mHz), high field precision ( ~ 10^−11^), and high-field (7T) operation, yielding advantages over counterpart NV sensors in this regime. This work opens avenues for NMR sensors at high fields, and opens new and interesting possibilities for employing dynamic nuclear polarization for quantum sensing.

## Methods

The diamond sample employed in this work is a single crystal CVD grown sample from Element6 with ~ 1ppm of NV centers. Initialization of the ^13^C nuclei via hyperpolarization is carried out via continuous laser illumination and chirped microwave excitation at low magnetic field (36–40 mT). The ^13^C magnetometry experiments here are all carried out at 7T. The spins are continuously interrogated in the windows between the *θ*-pulses, and digitized by means of a fast arbitrary waveform transceiver (Tabor Proteus). This allows us to measure the amplitude and phase of the ^13^C Larmor precession signal, from which we extract the transverse Bloch sphere components of the spin vector in the rotating frame. The AC field is applied by means of a $\hat{{{{{{{{\bf{z}}}}}}}}}$-coil parallel to the ^13^C quantization axis at 7T. More details about all these aspects, including instrumentation, protocols, and additional data are presented in the supplementary information.

## Supplementary information

Supplementary Information

## Data Availability

The data necessary to replicate the results of this paper are available from the corresponding author upon reasonable request.

## Code availability

The code required to reproduce the graphs and analysis in this paper are available in this Berkeley Box file sharing repository: https://berkeley.box.com/s/41ntv00tg5htvtqged3boyli5tn04q47.
