# Supplementary material for: High field magnetometry with hyperpolarized nuclear spins
Source: Nat Commun. 2022 Sep 19;13:5486. doi: 10.1038/s41467-022-32907-8 (PMC9485171; doi:10.1038/s41467-022-32907-8)
Supplement: Supplementary file 1 — Supplementary Information [file 41467_2022_32907_MOESM1_ESM.pdf]

Supplementary Information

High field magnetometry with hyperpolarized nuclear spins

O. Sahin[1], E. Sanchez[1], S. Conti[1], A. Akkiraju[1], P. Reshetikhin[1], E. Druga,[1] A. Aggarwal,[1] B. Gilbert[2], S. Bhave[3] and A. Ajoy[1,4]

[1]*Department of Chemistry, University of California, Berkeley, Berkeley, CA 94720, USA.*
[2]*Energy Geoscience Division, Lawrence Berkeley National Laboratory, Berkeley, CA 94720, USA.*
[3]*OxideMEMS Lab, Purdue University, 47907 West Lafayette, IN, USA.*
[4]*Chemical Sciences Division Lawrence Berkeley National Laboratory, Berkeley, CA 94720, USA.*

CONTENTS

References 8

Supplementary Note 1. Scaling of primary and secondary harmonic intensities 2

Supplementary Note 2. NMR Probe 2

Supplementary Note 3. Experiments probing coil cross-coupling 2

Supplementary Note 4. Data Processing 3

Supplementary Note 5. Phase unwrapping protocol 4

Supplementary Note 6. Hyperpolarization Setup and Mechanism 4

Supplementary Note 7. Fourier analysis of tracked signal magnetometry in Fig. 7 4

Supplementary Note 8. Average Hamiltonian Analysis 5

Supplementary Note 9. Spin evolution for off-resonance fields 6

Supplementary Note 10. Simulation of Sequence Performance 7
A. Dipolar Network Simulation 7
B. Delta Pulse Simulations 9

Supplementary Note 11. Estimation of sensitivity 9

Supplementary Note 12. Comparison between NV and ^{13}C magnetometers 9

Supplementary Note 13. Comparison to other high-field magnetometers 10

Supplementary Note 1. SCALING OF PRIMARY AND SECONDARY HARMONIC INTENSITIES

In this section, we present extended data on how Fig. 4D of the main paer was measured, demonstrating how relative scaling of signal intensities between the first and second harmonics changes with respect to pulse width t_p. We perform in Supplementary Figure 1 a series of experiments with conditions similar to Fig. 6, conducting AC field chirps in a 1-4 kHz window ($\Delta\mathcal{B}$=3 kHz) in 20 s. Here, we varied pulse width while fixing the acquisition windows at t_{acq}=32 μs. Dead times between pulses and acquisition were also kept constant. Following Eq. (1) then, the resonance condition shifts with t_p. This is evident in Supplementary Figure 1A, where we plot the zoomed response of the first harmonic (red) and second harmonic (blue dashed) respectively, while normalizing the peak of the second harmonic profile. We observe that the relative strength of the second harmonic intensity increases with pulse duty cycle.

From Supplementary Figure 1A, the resonance frequency is obtained from the cusp of the primary harmonic response (pink line), and also corresponds to half the frequency at which the second harmonic response (dashed blue line) is maximum. Supplementary Figure 1B (same as Fig. 4D in the main paper) plots this precise measurement of the resonance frequency f_{res} for differing pulse sequence parameters. Data points show a good fit to the expected dependence of the resonance frequency (Eq. (1)), wherein we extract $\theta=\pi/2$ for t_p=36μs. Supplementary Figure 1C elucidates the extracted ratio of the second to first harmonic intensities. The data indicates that the second harmonic response is related to finite pulse widths employed and will be absent in the limit of δ-pulses.

Supplementary Note 2. NMR PROBE

We now provide details of the NMR probe employed in the high field magnetometry experiments at 7 T. For this, we designed and built an NMR probe (see Supplementary Figure 2A-B) that:

(i) is capable of high fidelity ^{13}C inductive detection at 75 MHz,

(ii) with high RF homogeneity, allows ~275k pulses to be applied to the nuclear spins at high power (~30 W) and high duty cycle (~50%, applied every 73 μs) (Fig. 2A), and

(iii) permits the application of a time-varying (AC) magnetic field simultaneous with the pulse sequence (Fig. 2A).

This is accomplished with a combination of RF and z-coils as shown in Supplementary Figure 2A. The RF coil employed for ^{13}C readout is in a saddle geometry and is laser-cut out of OFHC copper with 3 turns and a coil height of 1cm. We measure a Q-factor of 30 at 75 MHz and a sample filling factor of ~0.15. The z-coil employed to apply the AC field is a loop of a 2-turn coaxial cable that minimizes electric fields (Supplementary Figure 2A).

Supplementary Figure 2C shows the circuits employed in these experiments. High SNR RF detection of the ^{13}C nuclear precession is obtained via a quarter wave line and bandpass filter combination following a transmit/receive switch, and the signal is digitized by a high-speed arbitrary waveform generator (Tabor Proteus). The high data acquisition rate of the device (1 GS/s) allows for high-fidelity sampling of the ^{13}C induction signal between the pulses. We refer the reader to Supplementary Note 4 for more details on data processing. The AC field is applied with a Rigol DG1022 signal generator, weakly amplified by an AE Techron 724 amplifier to provide sufficient current swing. This is useful in the experiments shown in Fig. 6 and Fig. 7 of the main paper, wherein frequency sweeps are employed to determine the frequency response of the sensor.

Supplementary Note 3. EXPERIMENTS PROBING COIL CROSS-COUPLING

We performed a series of experiments to ensure that the applied z-coil signals are not picked up by the RF coil. This would eliminate the possibility that the oscillatory ^{13}C dynamics in Fig. 5 arise from coil cross-coupling. We note at the outset that such pickup is expected to be negligible because:

i. The RF and z-coils are orthogonal to <1° and have little mutual inductance coupling, and

ii. The applied AC fields are in the 10 Hz-10 kHz range, far outside the detection range of the NMR RF circuit (tuned to 75 MHz±30 kHz). These applied AC signals are strongly suppressed by the quarter wave line and bandpass filters in the circuit, which deliver a >80 dB suppression.

Simple experiments bear out this intuition (Supplementary Figure 3). In these measurements, we applied a 1.75 kHz AC field of 100 mVpp amplitude with the Rigol signal generator and found the corresponding signal in the output under the following conditions:

i. In Supplementary Figure 3A, we removed the sample completely from the probe. The pulses (as in Fig. 2A of the main paper) were then applied and the resulting data was processed by the data handling pipeline shown in Fig. 2. We only observed noise in these measurements (Supplementary Figure 3A) and a Fourier transform (right panel) did not reveal any signals corresponding to the applied AC field; instead, we only observed noise.

ii. In Supplementary Figure 3B, we applied a test signal at 75 MHz with an additional x-coil, referred to here as the "external coil", in order to mimic a spin precession signal from the ^{13}C nuclei. The sample was still absent from the probe in these measurements. As expected, the experimental data reveals a flat non-decaying signal, and a Fourier transform contains a multitude of peaks due to noise. However there are no peaks at 1.75 kHz and 3.5 kHz, the expected positions of the first and second harmonics of the applied AC field. These are marked by the orange dashed lines, and the insets in Supplementary Figure 3B show a zoom into these regions, showing only noise.

iii. In Supplementary Figure 3C, we performed an experiment with the diamond sample thermally polarized for 10 s with no hyperpolarization. Once again, we only observed noise with no signature of the applied AC field.

iv. In Supplementary Figure 3D, the diamond sample was hyperpolarized, resulting in Fourier peaks at 1.75 kHz and 3.5 kHz characteristic of the applied AC field.

Therefore, we conclude that the AC field harmonics are obtained as a combined action of the spin-lock pulse sequence with the AC field on the hyperpolarized ^{13}C nuclei. This conclusion is strengthened by the fact that the effective frequency response of the ^{13}C sensor is not constant, and depends on the exact pulse spacing (see Fig. 7 of the main paper) resulting in a sharp response near the resonance condition.

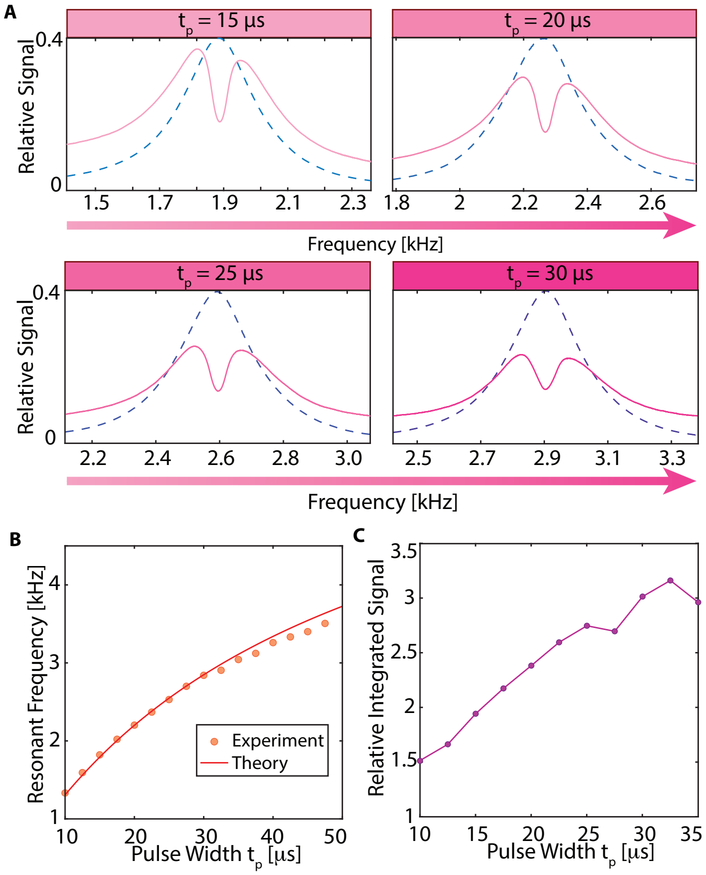

Supplementary Figure 1. **Scaling of primary and secondary harmonic intensities.** (A) Zoomed in windows corresponding to the primary (pink line) and secondary (dashed blue line) harmonic frequency response for pulsed spin-lock protocol with varying pulse duty cycle. Traces are normalized to second harmonic peak. While the frequency response remains identical, relative strength of the primary harmonic with respect to secondary harmonic decreases with increasing pulse duty cycle (arrow). (B) Scaling of the ^{13}C magnetometer resonance frequency with respect to interpulse spacing, estimated experimentally by halving the second harmonic peak frequency. Lines show theoretically predicted resonance frequencies at different applied flip angles. There is a good agreement with the theoretical prediction Eq. (1) (solid line). (C) Relative magnitude of the secondary harmonic intensity to the primary harmonic intensity, obtained from (A). Data indicates a redistribution of signal power to the second harmonic with increasing pulse width.

Supplementary Note 4. DATA PROCESSING

The data processing pipeline employed in this manuscript follows a similar approach as described in Ref. [31]. Here, we highlight salient features and emphasize the differences. The NMR signal is sampled continuously in $t_{\rm acq}$ windows between the pulses (Fig. 2A), at a sampling rate of 1GS/s via the Tabor Proteus. In typical experiments as in Fig. 2A, the pulses are spaced apart by τ=73 μs and the acquisition windows are $t_{\rm acq}$=32 μs. The spin precession is heterodyned to 20 MHz which is the oscillation frequency sampled in the measurements (as shown in Fig. 2C). For each acquisition window, we take a Fourier transform and extract the 20MHz peak as in Fig. 2 of the main paper. This corresponds to the application of a digital bandpass filter with a linewidth of $t_{\rm acq}^{-1}\approx$31.2kHz. For the 20 s long acquisition periods employed in the paper and a pulse spacing of 73 μs, we have ~275k data collection windows.

In the experimental data, we focus on two complementary aspects (highlighted in Fig. 3 of the main paper):

(i) The decay of the pulsed spin-lock signal as the AC field approaches the resonance condition.

(ii) Oscillations riding on the pulsed spin-lock signal, which carry the imprint of the AC field applied to the nuclei.

To isolate the decay (i), we smooth the pulsed spin-lock data over an interval of 73ms. This corresponds to a 13.7 Hz digital low-pass filter that suppresses the oscillations. Subtracting the smoothed data from the raw data curve reveals the oscillations (ii).

We emphasize that the ^{13}C oscillatory dynamics manifest as an amplitude modulation and not from the frequency shift of the spin precession outside the detection window. For example, Fig. 2B-D of the main paper shows the raw data obtained between the pulses and their corresponding Fourier transforms for the signal from an AC frequency of 2 kHz and a voltage intensity of 100 mVpp. Evidently, the AC field intensity is so low that it does not cause a shift in the Fourier transform peaks which remain at 20 MHz. To appreciably shift the frequency here, the AC field would have to be at least 30 G in strength — the fields we employ are two orders of magnitude lower. There is, instead, an amplitude variation between the FT peaks which is plotted in Fig. 2D of the main paper (oscillations which imprint the amplitude and frequency of the applied AC field).

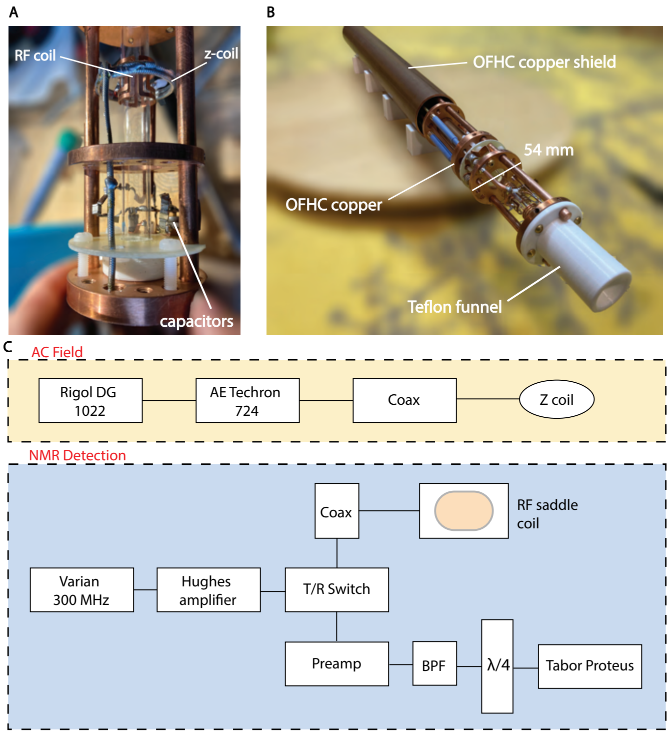

Supplementary Figure 2. **NMR Probe** used in experiments. (A) Photograph of internal probe components showing (i) RF coil used for ^{13}C NMR and (ii) z-coil by which the test AC field is applied. Both coils connect to independent rigid coaxial cables, but share a common ground. The diamond sensor is held under water in a test tube and is shuttled into the center of the RF coil (marked). (B) Zoomed out picture of probe highlighting its oxygen free (OFHC) copper construction, surrounded by a 54 mm OFHC copper shield (marked). (C) Circuit. Panel displays circuit employed in the experiments. The orange region shows circuit used for AC field application. The blue region shows NMR excitation and detection circuit.

Supplementary Note 5. PHASE UNWRAPPING PROTOCOL

Here we elucidate the phase unwrapping protocol employed for the extraction of the Fourier transform of the ^{13}C sensor response in each readout window in Fig. 2C of the main paper. This is employed in the sensitivity analysis (Supplementary Figure 7). First note that amplitude and phase at the heterodyned ^{13}C Larmor frequency report on the components of the spin vector along the equatorial $\hat{\mathbf{x}}$-$\hat{\mathbf{y}}$ plane. Consider Supplementary Figure 4C that illustrates this amplitude and phase data obtained in a 25.3 ms time window for two exemplary cases: (i) first, in the absence of the AC field (green line), and (ii) second, with an applied AC field on resonance at 1.953 kHz. In both cases, we employ a train of $\pi/2$ pulses. In the former case, the constant amplitude signal $S(t)$ (green) reflects the spins being locked stationary along the $\hat{\mathbf{x}}$ axis. However, when considering the phase, (Supplementary Figure 4B) ramps arise due to a trivial phase accrual under Larmor precession during each theta-pulse. Removal of this trivial phase is accomplished via a linear fit. This allows us to extract the effective phase evolution in the rotating frame (dressed by the AC field). Combining information from the two quadratures then allows direct access to the transverse projections I_x and I_y in the rotating frame. These projections are displayed in Supplementary Figure 4C(i-ii) for the resonant case considered, wherein the oscillations reflect a simple precession of the spins in the dressed frame.

Supplementary Note 6. HYPERPOLARIZATION SETUP AND MECHANISM

The hyperpolarization scheme we use in this work utilizes NV centers for transferring polarization to the ^{13}C nuclei. The diamond is subjected to a bias field of 38 mT. The NV center is optically hyperpolarized using a 520 nm laser. A MW chirp is applied at the same time which transfers polarization from the NV center to the ^{13}C nuclei with each chirp. After 40 s, the diamond is physically shuttled up to high-field at 7 T within a second.

The 520 nm 1W lasers are applied through a spherical "laser dome" so that the sample is irradiated approximately isotropically. In our experiments, we have used up to 30 lasers. The bias field is a combination of a current applied through a Helmholtz coil driven at 3.6 A in constant current mode and the fringe fields from the high-field magnet. The MW chirp sweep is digitally created and applied by a Arbitrary Waveform Transceiver (AWT) (Tabor Proteus) with a sampling frequency of 9 GS/s. The chirp is centered at 3.775 GHz with a chirp bandwidth of 24 MHz and the sweep frequency is 750 Hz. The MW then passes through an amplifier with a saturation power of 100 W and is then delivered to the sample through a MW coil around the sample. In our experiments, the MW power was around ~30 W.

For simplicity, it is easiest to think about the mechanism of transfer in a model system of an NV-^{13}C pair. The 520 nm laser optically hyperpolarizes the NV center into the m_s=0 state through a non-radiative relaxation pathway of the m_s=±1 levels of the electronic excited state triplet. The 38 mT bias field breaks the degeneracy of the m_s=±1 states of the NV center ground state triplet which allows selectively exciting only one of the levels. In our experiments, we are interested in the transitions between m_s=$\{0, +1\}$ levels. A combination of the MW drive and the hyperfine couplings create a pair of LZ anticrossings that are selectively adiabatic or diabatic depending on the starting nuclear state. The application of the MW chirp then creates a population imbalance between the nuclear spin levels resulting in hyperpolarization over multiple cycles of the MW chirp. A more detailed account of the mechanism can be found in [36].

Supplementary Note 7. FOURIER ANALYSIS OF TRACKED SIGNAL MAGNETOMETRY IN FIG. 7

In order to highlight aspects of the tracked signal magnetometry in Fig. 7 of the main paper, Supplementary Figure 5 shows the corresponding short-time Fourier transform of the data in Fig. 7A. This is represented as a 2D color plot by partitioning the time domain data from Fig. 7A into 500 windows (Δt=40ms and Fourier transforming each window. Two bright straight lines in the frequency response highlight the two harmonics of the ^{13}C magnetometer response, and demonstrate that they change linear with the applied AC field.

The increased intensity around resonance, corresponding to ≈2.7 kHz for the first harmonic and 5.4 kHz for the second harmonic is consistent with Fig. 6A. The measurement SNR is sufficient to resolve most of the frequencies in the 1-4 kHz band.

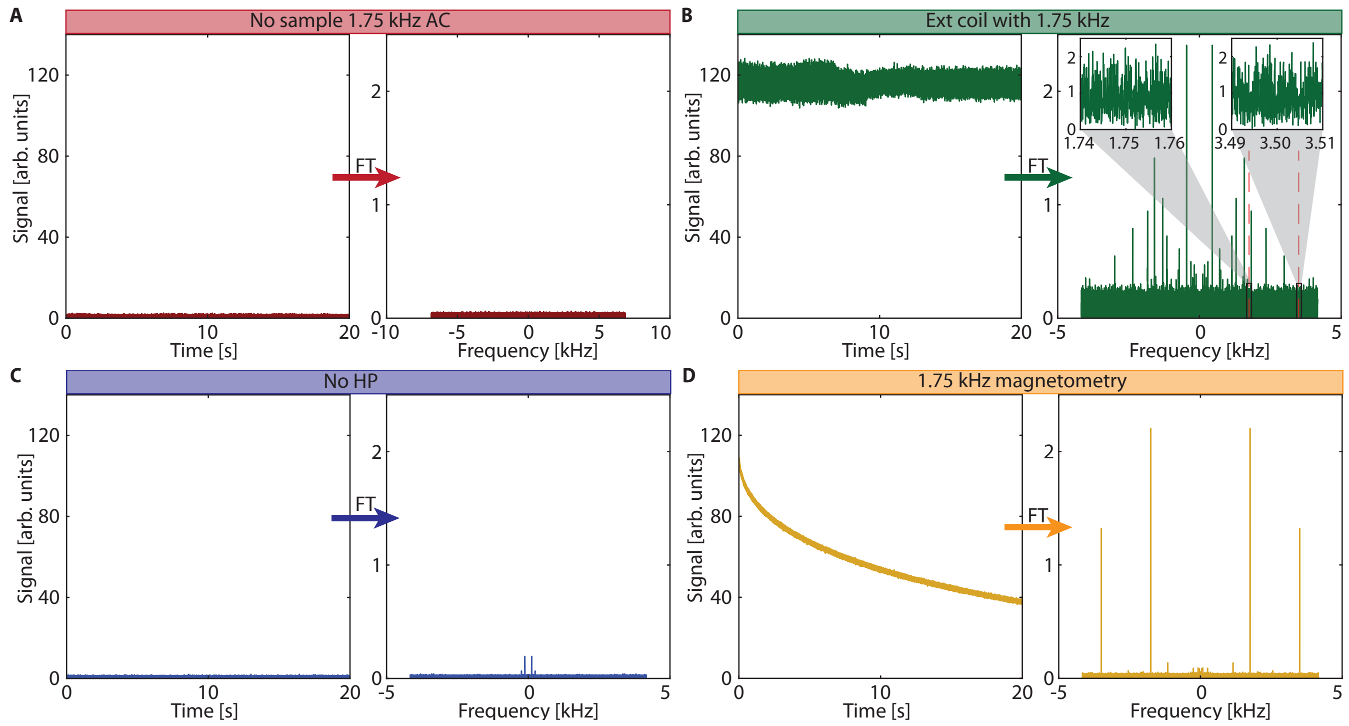

Supplementary Figure 3. **Experiments testing cross-coupling** between NMR detection and AC field coils. Panels show single-shot pulsed spin lock data and Fourier transforms of S_o (as shown in Fig.2C of the main paper). We apply a 1.75 kHz AC field in each experiment. (A) Data with no sample in probe. Signal obtained is just noise and Fourier transform shows no signatures of the AC field. Small peaks arise due to noise from probe. (B) Data with simulated field at 75 MHz applied by an additional x-coil, which we refer to as the external coil, with no sample in probe. This simulates precession of ^{13}C nuclei for the NMR receiver. Time domain data shows a flat non-decaying signal. Fourier transform shows no components at the applied AC frequency. Dashed orange lines indicate the frequencies at which we would expect to see the first and second harmonics. Inset: Zoomed into these regions for clarity. Signals at 1.75 kHz and 3.5 kHz are absent. (C) Data with diamond thermally polarized after 10 s in the magnet. Weak SNR without hyperpolarization makes AC field components indiscernible. (D) Reference data performed with hyperpolarized sample (corresponding to Fig. 5 of the main paper) showing time domain oscillations and Fourier peaks at 1.75 kHz.

The first harmonic is entirely in the first Nyquist zone (frequencies up to 6.849 kHz) though the second harmonic ends up in the second Nyquist zone for the higher frequencies. It is possible to observe an alias of the second harmonic near the 16 s mark.

Supplementary Note 8. AVERAGE HAMILTONIAN ANALYSIS

Here we present a more detailed average Hamiltonian analysis for the operation of the magnetometry sequence. For the majority of this section, unless otherwise explicitly stated, we treat the system in the lab frame. The total Hamiltonian of the system is $\mathcal{H}=\mathcal{H}_Z+\mathcal{H}_{dd}+\mathcal{H}_{AC}$ where $\mathcal{H}_Z=\omega_L I_z$ is the Zeeman Hamiltonian, $\mathcal{H}_{dd}=\sum_{k<\ell} b_{k\ell}(3I_{kz}I_{\ell z}-\vec{I_k}\cdot\vec{I_\ell})$ corresponds to the dipolar interaction, $\mathcal{H}_{AC}=\gamma_n B_{AC}\cos(2\pi f_{AC}t+\varphi_0)I_z$ is applied field to be sensed, $\omega_L = \gamma_n B_0$ is the Larmor frequency, and B_{AC}, w_{AC}, and φ_0 are the applied AC field amplitude, frequency, and phase respectively.

We will simplify the dipolar Hamiltonian, separate from the rest of the terms, using average Hamiltonian theory (AHT) by only keeping the leading-order term. These assumptions are reasonable under the condition $\zeta=2\pi J\tau\ll 1$. For each pulse period, we will treat the system in the toggling frame defined by the pulses up to that point. For the period after the jth pulse, the toggling frame transformation is given by

$$\mathcal{H}_{dd}^{(j)} = \exp\left(-ij\theta I_x\right)\mathcal{H}_{dd}\exp\left(ij\theta I_x\right), \qquad (4)$$

Consider the case when $\theta=\frac{\pi}{2}$. In this case, the toggling frame wraps to the original frame after a period of 4τ. For a multiple period of 4τ then, the system dynamics is captured by the average Hamiltonian,

$$\mathcal{H}_{dd}^{(0)} = \sum \mathcal{H}_{dd}^{(j)} = \sum_{j<k} b_{kl}\left(\frac{3}{2}(I_{jz}I_{kz}+I_{jy}I_{ky})-\vec{I_j}\cdot\vec{I_k}\right). \qquad (5)$$

Higher order AHT terms are evaluated in detail in Ref. [34]. The initial state $\rho(0)=I_x$ is protected against dipolar coupling because $[\mathcal{H}_{dd}^{(0)}, \rho(0)]=0$. Since the state is protected over an average, we will neglect the dipolar term in all of the following sections.

We now treat the external field Hamiltonian, H_{AC} using the same method, once again assuming $\theta=\pi/2$. A DC field, whose lab frame Hamiltonian is given by $H_{AC}=\gamma_n B I_z$, will be rotated by $\pi/2$ by each toggling frame transformation such that over a four pulse period, the Hamiltonian will average to 0. It is possible to map the toggling frame Hamiltonians for each pulse period on a phasor in order to see this more clearly (Fig. 4C). For this case, the toggling frame Hamiltonians will cover the phasor symmetrically such that the vectoral sum on the phasor will be 0. This also works for the more general case $\theta=\frac{2\pi k}{n}$ for integers k and n, as the points will trace a regular polygon on the phasor, which will still vanish when summed.

However, for a resonant AC field (assumed to be a square wave for simplicity) with frequency $f_{AC}=1/4\tau$, the average Hamiltonian is a linear combination of I_y and I_z (for the special cases where the AC field phase is $\varphi=-\frac{\pi}{6}$ or $\frac{5\pi}{6}$, the I_z terms cancel

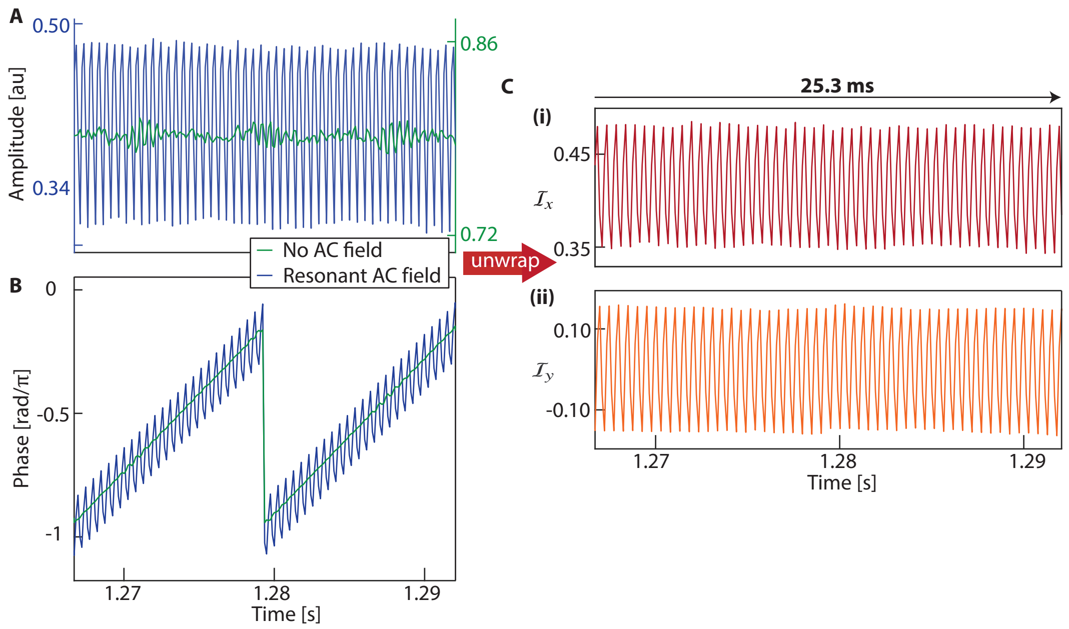

Supplementary Figure 4. **Phase unwrapping protocol** (A-B) Amplitude and phase response for a representative 25.3 ms window under: (i) no applied AC field (green), and (ii) a resonant AC field at 1953.150 Hz (blue). Sequence here employs $\pi/2$ pulses. Small wiggles in the green line are due to 60 Hz noise pickup. Ramp-like phase pattern indicates phase accrued during pulses. (C) Rotating frame Cartesian coordinates trajectory components extracted for (ii) above.

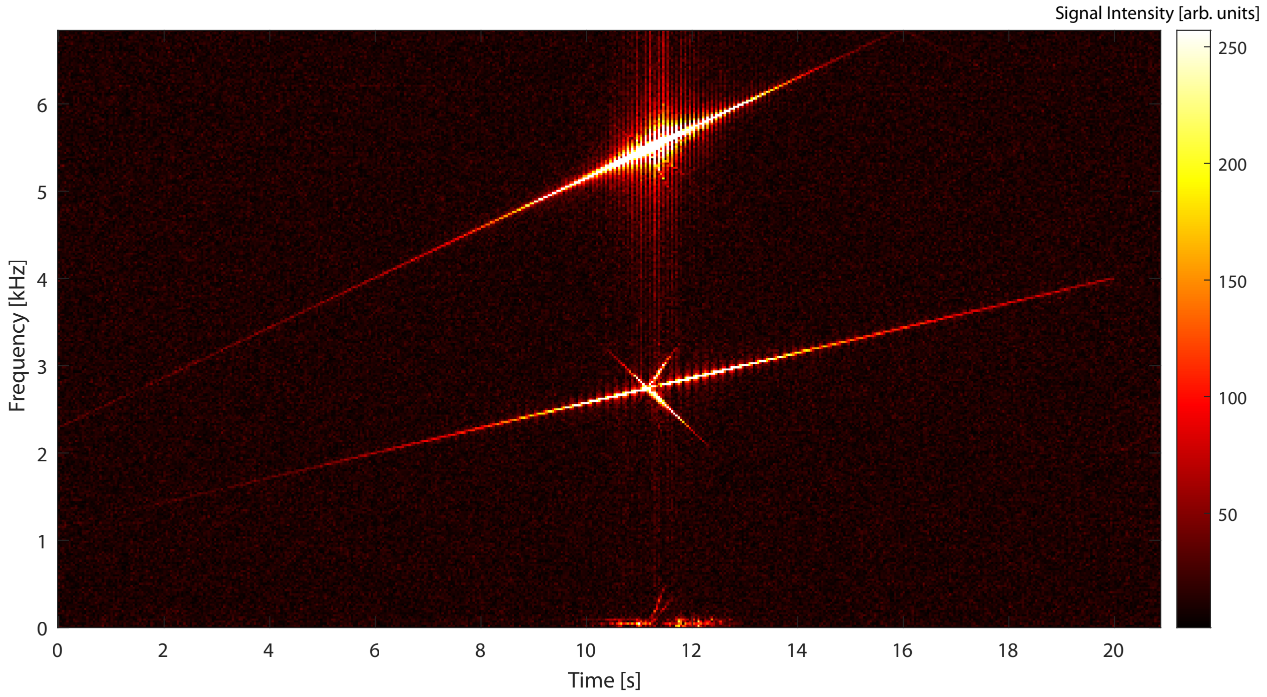

Supplementary Figure 5. **Fourier representation of tracked magnetometry** in Fig. 7 of the main paper. Here a Fourier transform of the time domain data (Fig. 7A) under a chirped AC field is taken every ~40 ms (500 total time windows). Colors represent intensity of corresponding spectral components up to the first Nyquist zone. Data reveals two lines corresponding to the first (lower) and the second (upper) harmonics respectively. Spectral intensity is strongest near resonance (≈2.7kHz (see Fig. 6B). Alias of the second harmonic can be seen after 15.8 s on the time axis.

out but there is a net I_y term). For any AC field with $f_{AC}{\neq}f_{res}$, the external field Hamiltonian will average to zero, albeit over a longer timescale.

Supplementary Note 9. SPIN EVOLUTION FOR OFF-RESONANCE FIELDS

In the main paper, we had elucidated theory of the experiment as a "rotating-frame" NMR analogue. This calculation was provided for the resonant case, i.e. $f_{AC}{=}f_{res}$. Sup-

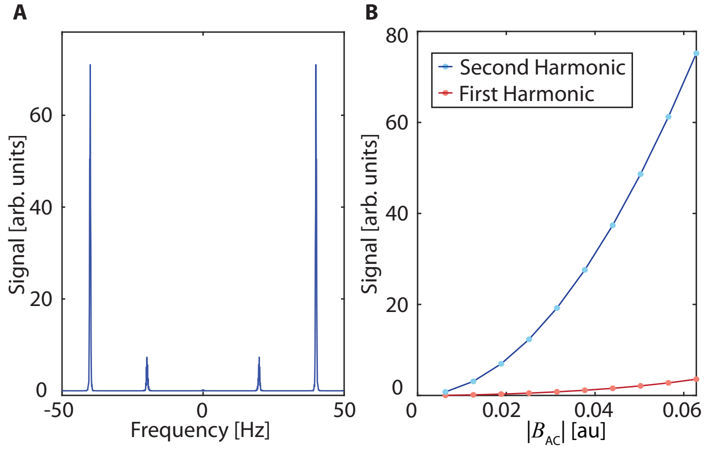

Supplementary Figure 6. **Simulation of the signal** in the resonant case f_{AC}=f_{res}=20Hz, following the theoretical expression derived in the main paper. (A) Left panel shows the magnitude of the Fourier transform of the oscillatory component S_o of the signal. It reveals two harmonics at f_{AC} and $2f_{AC}$ respectively. (B) Right panel shows the scaling of the magnitudes of the two harmonics, and shows good qualitative agreement with the experimental results in Fig. 5H.

plementary Figure 6 shows a simulation of the signal $S(t) = \left[\cos^2(\gamma_n B_{AC} t)+\sin^2(\gamma_n B_{AC} t)\sin^2(2\pi f_{AC} t)\right]^{1/2}$, obtained for a representative example of f_{AC}=20Hz. Plotted is the magnitude of the Fourier transform of the oscillatory signal S_o similar to Fig. 3C and Fig. 5 of the main paper. The Fourier transform in Supplementary Figure 6A reveals the precedence of two harmonics (primary and secondary). Supplementary Figure 6B plots the magnitudes of both harmonics, which is in good qualitative agreement with the data in Fig. 5H.

In this section, we extend this calculation to the off-resonant case, i.e. for arbitrary applied frequency f_{AC}. Here we neglect the effect of dipolar interaction driven evolution, assuming it is suppressed via the analysis above. Consider first that the rotating frame Hamiltonian (at ω_L) can be written of the form,

$$\mathcal{H} = \Omega I_x + \gamma_n B_{AC}\cos(2\pi f_{AC} t) I_z \quad (6)$$

In a second rotating frame at $2\pi f_{AC}$ and assuming a rotating wave approximation $\gamma_n|B_{AC}| \ll f_{AC}$, one can write down the state evolution as,

$$\rho'_R(t) = \cos\alpha\cos(Qt)I_x + \cos\alpha\sin(Qt)I_y + \sin\alpha I_z, \quad (7)$$

where $\tan\alpha = (\Omega - 2\pi f_{AC})/(\gamma B_{AC})$, and $Q = \cos\theta\left[(\Omega-2\pi f_{AC})\tan\alpha + \frac{1}{2}\gamma B_{AC}\right]$. In the original rotating frame, the state then has the form,

$$\rho_R(t) = \left(\cos^2\alpha\cos(Qt)+\sin^2\alpha\right)I_x + \cos\alpha\sin(Qt)I_y. \quad (8)$$

Finally returning to the lab frame where the measurement is carried out, yields the state evolution,

$$\rho(t) = \left(\cos^2\alpha\cos(Qt)+\sin^2\alpha\right)I_x + \cos\alpha\sin(Qt)\cos(2\pi f_{AC} t)I_y + \cos\alpha\sin(Qt)\sin(2\pi f_{AC} t)I_z. \quad (9)$$

Ultimately, since we measure $S = [\langle I_x\rangle^2 + \langle I_y\rangle^2]^{1/2}$, there is an oscillation of the form,

$$S = \left[\left(\cos^2\alpha\cos(Qt)+\sin^2\alpha\right)^2 + \cos^2\alpha\sin^2(Qt)\cos^2(2\pi f_{AC} t)\right]^{1/2} \quad (10)$$

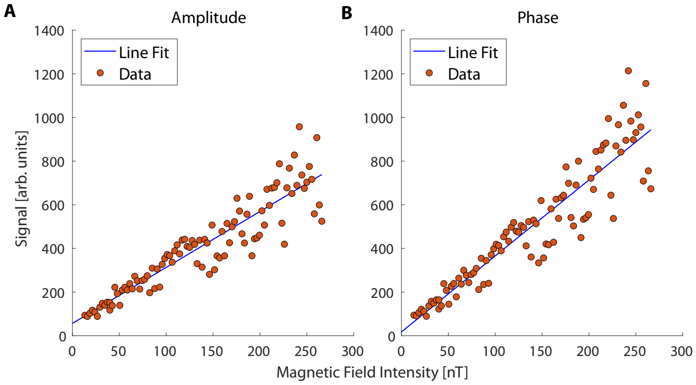

Supplementary Figure 7. **Sensitivity analysis.** Points show the measured strength of the first harmonic of the oscillatory signal for varying strengths of the AC field $|B_{AC}|$ obtained via (A) the magnitude and (B) phase of the FT signal as in Fig. 2 of the main paper. Data here is carried out for t=34s of signal acquisition. For each case, the noise of the added AC field manifests as scattered data points for larger $|B_{AC}|$. This does not reflect the sensitivity of the ^{13}C sensor. A linear fit gives an overall estimate of sensitivity, but an extrapolation of the data to zero cannot be made. An overall estimation of the sensitivity is done by finding the uncertainty associated with each point and calculating the corresponding change in the magnetic field strength using the slope of the fitted line. The reported sensitivity is the mean of all of the sensitivities for each point.

that carries first and second harmonics of f_{AC}, similar to Supplementary Figure 6. Note that this picture is a simplified model for the system dynamics that does not take certain multi-body effects into account and as such, we would only expect it to predict certain qualitative aspects of this phenomenon such as the harmonics in the frequency response. A more detailed and complete account of this phenomenon will be the subject of a future work.

Supplementary Note 10. SIMULATION OF SEQUENCE PERFORMANCE

A. Dipolar Network Simulation

For the result in Fig. 4B, we numerically simulate a sequence operation assuming a Hamiltonian of the form, $\mathcal{H} = k_{dd}\mathcal{H}_{dd} + k_z\mathcal{H}_z$ where $\mathcal{H}_{dd}$ and $\mathcal{H}_z$ are both normalized such that, $\|\mathcal{H}_{dd}\| = 1$, where $\|.\|$ refers to the Frobenius norm.

We assume application of a square-modulated AC magnetic field at frequency f_{AC}. We consider the average results from 10 random configurations of a N=5 spin ^{13}C nuclear network, and simulate dynamics under the full spin Hamiltonian $\mathcal{H}$. Let t_j denote "switching events", i.e. time instants where either a pulse is applied, or there is a sign switch in the AC field. The propagator then evaluates to, $U = \prod_j U_j$, where $U_p = \exp\left[i\left(k_{dd}\mathcal{H}_{dd} + k_z H_z s_j\right)\Delta\tau_j\right]$, and $\Delta\tau_j = (\tau_{j+1} - \tau_j)$, and s_j is a sign term with s_1=1. We have (i) in case of a pulse event, $U_j = U_p e^{i\theta I_x}$, with $s_{j+1} = s_j$; (ii) upon a field sign switch $U_j = U_p$, with $s_{j+1} = -s_j$; and (iii) when both occur simultaneously, $U_j = U_p e^{i\theta I_x}$, with $s_{j+1} = -s_j$. The sequence fidelity is then evaluated via the survival probability in the $\hat{\mathbf{x}}$-$\hat{\mathbf{y}}$ plane.

In order to obtain the linewidth scaling with respect to the number of pulses as shown in Fig. 4G, we run this simulation for different numbers of pulses (all multiples of 4) and the resulting

Category	Parameter	Single NV[a,b]	Subensemble NV[c]	Nanodiamond NV[d]	Bulk Crystal NV[e]	**^{13}C Crystal**
Spin properties	T_1	7.4 ms[a], ≈5 ms[b]	3 ms	~1 ms[g]	~1 ms[f]	***1518 ± 20 s*** [j]
	T_2^*	1.54 ms[a], ≈5 μs[b]	750 ns	~1 μs[g]	~1 μs[f]	***1.5 ms*** [j]
	T_2	2.43 ms[a], 1.8 ms[b]	6.5 μs	35 μs	50 μs	***90.9 s*** [j]
Sensor spins	Sensor concentration	$3\text{x}10^{17}$ cm^{-3}[a], 10^{10} cm^{-3}[b]	3×10^{17} cm^{-3}	6×10^{17}cm^{-3}[h]	2×10^{17} cm^{-3}	2×10^{21} cm^{-3}
	Sensor spin coupling <d>	NA	51 kHz	103 kHz	26 kHz	660 Hz
	Sensor interaction figure-of-merit <d>T_2^*	NA	0.039	~0.1	0.026	1
	Sensor volume	Single spin	1021 μm^3	1.4×10^4 nm^3	8.7×10^5 μm^3	~3.6 mm^3[l]
Sensor properties	Sensitivity	9.1 nT/√Hz[a], 4.3 nT/√Hz[b]	50 pT/√Hz	0.1 μT[i]	0.9 pT/√Hz	410 nT/√Hz
	Bandwidth	>10 MHz[f]	≈41 kHz	2 MHz	20 kHz	8 kHz
	Frequency resolution	≈556 kHz[b]	<1 mHz	29 kHz	6 kHz	2.2 mHz
	Precision	6.2×10^{-8} /√Hz[b]	5.7×10^{-10} /√Hz	4.1×10^{-6} /√Hz	2×10^{-10} /√Hz	5.8×10^{-11} /√Hz
	Operating field	1.8 mT[a], 69 mT[b]	88 mT	24.2 mT	4.6 mT	7 T[k]
Sensor interrogation	Projective/ continuous	Projective				Continuous
Special niches	Transportable	No				Yes
	Scattering media	No				Yes
	High field operation	No				Yes

(a) Herbschleb, E.D. et al. Ultra-long spin coherence times amongst room-temperature solid-state spins. Nat. Commun. 10, 3766 (2019). **(b)** Balasubramanian, G. et al. Ultralong spin coherence time in isotopically engineered diamond. Nat. Mater. 8, 383-387 (2009). **(c)** Glenn, D. et al. High-resolution magnetic resonance spectroscopy using a solid-state spin sensor. Nature 555, 351–354 (2018). **(d)** Holzgrafe, J. et al. Nanoscale NMR Spectroscopy Using Nanodiamond Quantum Sensors. Phys. Rev. Applied 13, 044004 (2020). **(e)** Wolf, T. et al. Subpicotesla Diamond Magnetometer. Physical Review X 5, 041001 (2015). **(f)** Value not reported in paper, estimate based on: Joas T. et al. Quantum sensing of weak radio-frequency signals by pulsed Mollow absorption spectroscopy. Nat. Comm. 8, 964 (2017). **(g)** Values were not reported in the papers, estimates based on existing literature. **(h)** Assuming 10% conversion from nitrogen**. (i)** Sensitivity was not reported as /√Hz. **(j)** Quoted here is the transverse spin lifetime as reported in: Beatrez, W. et al. Floquet prethermalization with lifetime exceeding 90s in a bulk hyperpolarized solid (2021). **(k)** Full range of magnetometer is 10 mT – 20 T. **(l)** Considering the single crystal sample with dimensions 3x3x1 mm and assumed penetration depth of 0.15 mm.

Supplementary Table 1. **Comparison between NV center magnetometers and ^{13}C nuclear sensors** considering representative literature for different sensor sizes. ^{13}C sensors have complementary properties to NV sensors and are attractive in some operating niches.

frequency response is fit to a gaussian profile peaking around the resonance frequency. The linewidth is then extracted as the FWHM of the fit and plotted as a function of the number of pulses (L). This process is then repeated for different dipolar coupling strengths ($k_{\rm dd}$).

In order to compare this numerical result with zeroth-order AHT, we run a similar simulation for AHT where instead of computing the propagator for each time event, we record the toggling frame "angle" for the time block between that event and the next. A pulse rotates the toggling frame by θ and a sign flip of the square wave rotates it by π. We then compute the average Hamiltonian for $\mathcal{H}_z$ to be $\overline{\mathcal{H}}_{\rm z}^{(0)} = \sum_j \exp(i\theta_j \Delta t_j)$ where j is the index for a given time block, θ_j is the toggling frame angle for that time block, and Δt_j is the length of that time block. The real part of the resulting complex number corresponds to the coefficient of the I_z term and the imaginary part, the $-I_y$ term. The dipolar term is treated similarly. The effect of a toggling frame rotation by θ on the dipolar Hamiltonian is given by,

$$\mathcal{H}_{\rm dd}^{(\theta)} = \sum_{j<k} \cos^2(\theta) I_{jz} I_{kz} + \cos(\theta)\sin(\theta)(I_{jy}I_{kz} + I_{jz}I_{ky}) + \sin^2(\theta) I_{jy} I_{ky}. \quad (11)$$

The time propagator then becomes $U = \exp(-i(\overline{\mathcal{H}}_{\rm dd}^{(0)} + \overline{\mathcal{H}}_{\rm z}^{(0)})t)$ where $t = \sum_j t_j$ is the total time run by the simulation and the fidelity is once again evaluated. Both simulations are then run for multiple AC frequencies in order to obtain the results for Fig. 4B.

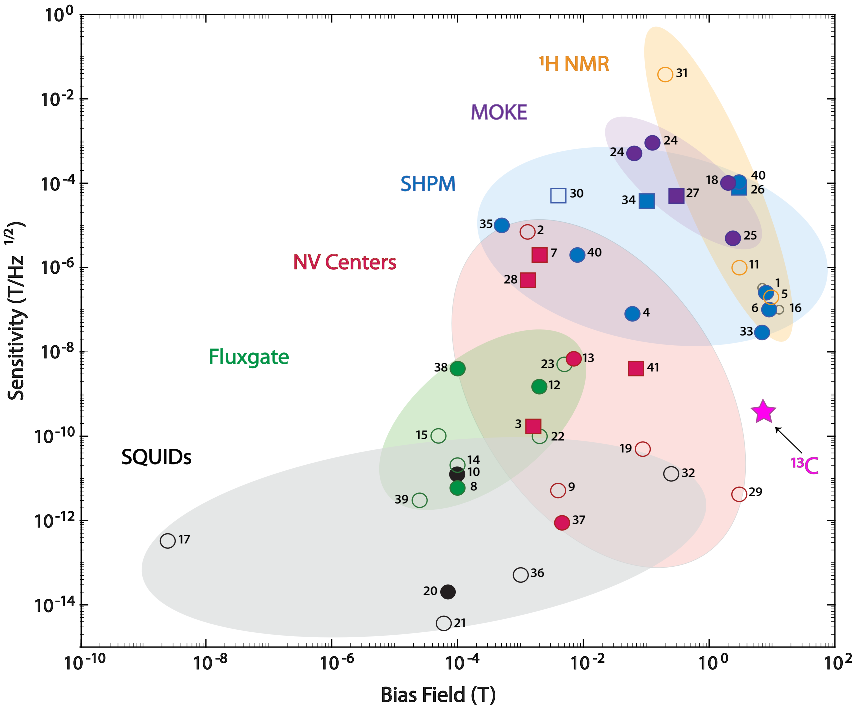

(1) Rostami et al. Instrum. Exp. Tech. 59, 2, 273-277 (2016). **(2)** Kehayias et al. J. Appl. Phys. 127, 203904 (2020). **(3)** Maletinsky et al. Nat. Nanotechnol 7, 320-324 (2012). **(4)** Gregory et al. Rev. Sci. Instrum. 73, 3515 (2002). **(5)** Handwerker et al. Biomed Tech 58 (2013). **(6)** Karcı et al. Rev. Sci. Instrum. 85, 103703 (2014). **(7)** Dréau et al. Phys. Rev. B 84, 195204 (2011). **(8)** GMW Associates, USA. **(9)** Acosta et al. Appl. Phys. Lett. 97, 174104 (2010). **(10)** Cryogenic Limited, UK. **(11)** Group3 Technology, New Zealand. **(12)** Digi-Key Electronics, USA. **(13)** Pham et al. Phys. Rev. B 86, 045214 (2012). **(14)** Smagall Measuring Equipment Co., China. **(15)** Lemi LCC, Ukraine. **(16)** Caylar SAS, France. **(17)** Baudenbacher et al. Rev. Sci. Instrum. 73, 1247 (2002). **(18)** Holmarc Opto-Mechanics PVT. LTD., India. **(19)** Glenn at al. Nature 555, 351 (2018). **(20)** Drung Supercond. Sci. Technol. 16, 1320-1336 (2003). **(21)** Drung et al. IEEE Trans. Appl. Supercond. 17, 699-704 (2007). **(22)** Bartington Instruments, UK. **(23)** Matesy GmbH, Germany. **(24)** Matesy GmbH, Germany. **(25)** Microsense - A KLA Company, USA. **(26)** Sandhu et al. Microelectron. Eng, 73-74, 524-528 (2004). **(27)** Quantum Design, Inc., USA. **(28)** Maze et al. Nature 455, 644-647 (2008). **(29)** Aslam et al. Science 357, 6346, 67-71 (2017). **(30)** Hicks et al. Appl. Phys. Lett. 90, 133512 (2007). **(31)** Metrolab Technology SA, Switzerland. **(32)** Quantum Design, Inc., USA. **(33)** Oral and Bending Appl. Phys. Lett. 69, 1324 (1996). **(34)** Sandhu et al. Jpn. J. Appl. Phys. 40, 4321 (2001). **(35)** Chang et al. Appl. Phys. Lett 61, 1974 (1992). **(36)** Tristan Technologies, USA. **(37)** Wolf et al. Phys. Rev. X 5, 041001 (2015). **(38)** Metrolab Technology SA, Switzerland. **(39)** Auster et al. Space Sci Rev 141, 235-264 (2008). **(40)** Metrolab Technology SA, Switzerland. **(41)** Balasubramanian et al. Nature Mater 8, 383-387 (2009).

Supplementary Figure 8. **Magnetometer landscape** focusing on a comparison with respect to sensitivity and bias field of operation. Points quote values from literature listed in footnote (right). Micro/nano sensors and macro sensors are depicted as squares and circles respectively. Filled points represent AC magnetic field sensors and outlined points represent DC sensors. ^{13}C magnetometers (depicted as a star) occupy a niche for high sensitivity magnetometry at high bias field.

B. Delta Pulse Simulations

The general spin trajectory under a $\pi/2$ pulse train as depicted in Fig. 1B is obtained by considering a single spin system initialized in the $\rho(0) = \cos(\frac{\pi}{6})I_x{+}\sin(\frac{\pi}{6})I_y$ state, a $\frac{\pi}{6}$ deviation from the $\hat{\mathbf{x}}$-axis and on the $\hat{\mathbf{x}}$-$\hat{\mathbf{y}}$ plane. The spin is under the previously mentioned pulse sequence and an external AC field resonant with that sequence ($f_{\rm AC}{=}f_{\rm res}{=}\frac{1}{4\tau}$) along the $\hat{\mathbf{z}}$-direction with an intensity of 16 nT. Our strategy is to numerically solve the classical Bloch equations for this system in the rotating frame. We achieve that by doing a finite element analysis with a time step such that each pulse period has 2000 points (~85 ns) and rotating the state along the $\hat{\mathbf{z}}$-axis by $2\pi\gamma_n B(t)\Delta t$ where $B(t)$ is the magnetic field intensity at time t and Δt is the timestep. If a pulse happens after a timestep, the state is rotated by $\pi/2$ along the $\hat{\mathbf{x}}$-axis. The simulation is run for 100 cycles of the AC field period and plotted on a Bloch sphere in order to obtain the graph in Fig. 1B.

Supplementary Note 11. ESTIMATION OF SENSITIVITY

To estimate the sensitivity of the ^{13}C sensor, we perform a careful calibration experiment with an AC fields of known intensity and compare against the strength of the signal response for the first harmonic of the obtained oscillatory signal. In particular, in these experiments, we apply an AC field via an calibrated current input through z-coil in Supplementary Figure 2. The current is measured via the voltage drop through a series resistor, and the field is estimated using Biot-Savart law.

We then measure the strength of the oscillatory signal for different values of $|B_{\rm AC}|$, measuring them via the magnitude and phase of the FT signal (see Supplementary Note 5) in Fig. 2 of the main paper. This is shown in Supplementary Figure 7. Measurements here are carried out in a single shot for t=34s. The phase signal concentrates all the intensity in the first harmonic and hence carries higher sensitivity. However, it needs to be unwrapped to extract only the phase of the spins in the rotating frame (removing the trivial phase accrued during the t_p pulse periods). We will detail a numerical procedure for this unwrapping, as briefly introduced in Supplementary Note 5, in a forthcoming paper. In each case, magnitude and phase, we estimate sensitivity by estimating the uncertainty associated with each point using the noise level of each data. We then calculate the corresponding change in the magnetic field intensity by doing a linear regression on the obtained signal and dividing each uncertainty by the slope of the plot. This allows us to discern the minimum signal that could be picked up by sensor at each point. The reported sensitivity is the mean of all of these calculated sensitivities. Through this, we estimate a smallest measurable signal in a single-shot as 130±22 pT and 70±15 pT respectively, yielding a sensitivity of 760±127 pT/$\sqrt{\rm Hz}$ and 410±90 pT/$\sqrt{\rm Hz}$ respectively at 95% CI. These are the results quoted in the main paper.

Supplementary Note 12. COMPARISON BETWEEN NV AND ^{13}C MAGNETOMETERS

Here we contrast the key features of ^{13}C nuclear spins and NV centers focused on applications in magnetometry. We include in this comparison four representative papers from the literature for different regimes of NV center quantum sensors - (i) single NV electrons well-isolated in the lattice (ii) sub-ensembles of NV

centers (occupying <100 μm^3) in single crystal samples, (iii) ensembles of NVs in microdiamond, and (iv) dense NV centers in bulk single crystal samples. For each, we elucidate values of key spin parameters, and in the last column in 1 compare them to corresponding properties of ^{13}C nuclear spins for the single crystal sample used in this work (same as that employed in Ref. [31]). Table (1) shows the complementary properties of NV electrons and ^{13}C nuclei for sensing. To make the comparison clearer, we focus on five major aspects as detailed below:

i. Spin properties: focusing on the respective values of T_2^*, T_2, and T_1. For T_2, we quote the values obtained pulsed driving (e.g. DD) that is relevant for quantum sensing. For the ^{13}C case, we quote the rotating frame lifetime value T_2' under pulsed spin locking [31]. This sets the total interrogation time that the ^{13}C sensors can permit. We qualify that this does not correspond to the total time over which the ^{13}C sensors can continue to accrue phase; which instead depends on an interplay between T_2^* and $f_{\rm AC}$, and is more difficult to estimate. We do emphasize however that for the ^{13}C case, the extension in the T_2' value, T_2'/T_2^* (≈60, 000 in [31]), is considerably larger than a naive scaling from the corresponding electron spin values by just the ratio of gyromagnetic ratios.

ii. Independent or coupled sensors: In the Table (1), we estimate the coupling strengths $\langle d\rangle$ between the quantum sensors in each of the samples considered. Even at natural abundance, the ^{13}C sensor concentration at least four orders of magnitude denser than NV center sensors. Evidently then, NV center quantum sensors largely operate in the limit of uncoupled (independent) sensors, wherein their T_2^* times are dominated by interactions with other spins as opposed to inter-sensor interactions. In contrast, ^{13}C sensors operate in the limit where interspin couplings, scaling with enrichment as $\eta^{1/2}$, dominate the T_2^* FID times. This can be seen by comparing the product $\langle d\rangle T_2^*$. Our pulsed spin-lock scheme is able to mitigate the effect of these couplings, allows interrogation up to T_2'.

iii. Sensor properties for AC magnetometry: focusing on sensitivity, bandwidth, spectral resolution, precision, and operating field. We note that while our experiments were carried out on a diamond crystal that was uniformly illuminated, we estimate a penetration depth <0.15mm [51]. Sensitivity refers to the smallest field that can be reproducibly measured over the bias magnetic field. Precision here refers to the magnetic field sensitivity by the bias field. In contrast to the lower field operation of the NV center quantum sensors, the ^{13}C sensor operates at 7 T. We estimate that the full dynamic range reaches can exceed 24 T, presenting the key strength of the approach here. Assuming a measurement up to 573 s (as demonstrated in Ref. [31]), a frequency resolution of 2.2 mHz is viable for the ^{13}C sensor, without reinitialization.

iv. Sensor interrogation: focusing on how in each case the sensors are prepared and read out. We note that ^{13}C readout is not projective and can proceed simultaneously with the magnetometry pulse sequence without reinitialization. Once the spins are polarized, the sensing period can last up to 10 min [31]. NV center sensors must be reinitialized before readout.

v. Special niches: Finally, in Table (1), we summarize some special features of the ^{13}C sensors. This can special niches for the operation of these sensors. First, the long T_1 time can potentially allow a separation between of field regions corresponding to ^{13}C initialization, sensing, and readout. More interestingly, the multiple-minute-long ^{13}C T_2' lifetimes means that the diamond sample can be continuously transported during the spin-lock pulses (within the RF coil). This has important implications for being able to up-convert DC fields of interest into AC fields that can be detected, an application that we wish to demonstrate in future work. Second, RF interrogation allows operation in optically dense or scattering media. Finally, their low gyromagnetic ratio allows operation at high magnetic fields.

Supplementary Note 13. COMPARISON TO OTHER HIGH-FIELD MAGNETOMETERS

Our ^{13}C sensors are particularly suited for the high-field magnetometry of time-varying signals. Particularly, their ability to obtain a high frequency resolution (<50 mHz) over a 7 kHz bandwidth in a wide range of magnetic fields is an important advantage over competing magnetometers. In this section, we compare ^{13}C sensors to common magnetometry techniques. We have included SQUID, Fluxgate, Scanning Hall Probe Microscopy (SHPM), Magneto-Optic Kerr Effect (MOKE), and ^{1}H NMR magnetometers in this comparison because of their ubiquity, and NV centers because of their close relation to ^{13}C sensors.

Supplementary Figure 8 graphically depicts a "landscape" of these sensor technologies, focusing on their sensitivity and bias field of operation. Sensitivity (y-axis) is defined as the smallest measurable field δB over the bias field B_0 (x-axis) and is smaller for better magnetic field sensors. Because sensitivity depends, in general, on sensor size, Supplementary Figure 8 draws a distinction between nano/microscale (squares) and macroscale (circles) sensors, as well as AC field sensors (filled) and DC field sensors (outlined). Shaded regions show representative values for each technology.

As shown in Supplementary Figure 8, there is currently an unmet need for high-field AC magnetometers with high resolution-to-bandwidth ratio. The ^{13}C sensor demonstrated in this work and illustrated by the pink star precisely fills this important niche. While our current work is carried out at 7 T, it is very straightforward to extend it to magnetic fields up to 24 T because of the slow-scaling ^{13}C gyromagnetic ratio. In this regime, we occupy a space with MOKE, SHPM, and ^{1}H NMR, but unlike these technologies, we are not restricted to bulk sensor sizes (^{1}H NMR) or DC and low frequency sensing. The sensitivity reported in this paper, 410 pT/$\sqrt{\rm Hz}$, is comparable to NV center and Fluxgate magnetometers, but also allows AC magnetometry with mHz resolution. We expect to further improve sensitivity with technical enhancements of our hyperpolarization and measurement apparatus. While ^{1}H NMR is currently the most popular technique at very high bias fields, the proposed ^{13}C magnetometer may serve as a replacement to these bulky sensors, with even higher sensitivity and polarization.
